# Genetic Association of Sexual Behavior, Sildenafil, and Hormonal Contraceptives With Mental Disorders

**DOI:** 10.1002/cns.70861

**Published:** 2026-04-16

**Authors:** Zhongliang Lin, Kejing Zhu, Renke He, Xueying Liu, Qinyu Luo, Haiyan Wu, Zhaoying Jiang, Jiaen Yu, Jianzhong Sheng, Hong Zhu, Hefeng Huang

**Affiliations:** ^1^ Key Laboratory of Reproductive Genetics (Ministry of Education), Department of Reproductive Endocrinology, Women's Hospital Zhejiang University School of Medicine Hangzhou China; ^2^ Reproductive Medicine Center, International Institutes of Medicine, the Fourth Affiliated Hospital Zhejiang University School of Medicine Yiwu Zhejiang China; ^3^ Research Units of Embryo Original Diseases Chinese Academy of Medical Sciences (No. 2019RU056) Shanghai China; ^4^ Obstetrics and Gynecology Hospital, Institute of Reproduction and Development Fudan University Shanghai China; ^5^ Shanghai Key Laboratory of Embryo Original Diseases Shanghai China

**Keywords:** contraceptives, eQTL, Mendelian randomization, mental disorders, NHANES, sexual behaviors, sildenafil

## Abstract

**Background:**

The observational research indicates that risky sexual activities contribute to the development of mental disorders. However, the potential causal effects of sexual behaviors, sildenafil use, and hormonal contraceptive use on mental disorders remain unclear.

**Methods:**

We applied two‐sample Mendelian randomization (univariable and multivariable MR) to assess the effects of age at first sex (AFS) and number of sexual partners (NSP) on mental disorders and sex differences, using Genome‐Wide Association Studies (GWAS) data from UK Biobank and FinnGen. An observational analysis using NHANES 2015–2016 was conducted for validation. We further used expression quantitative trait loci (eQTL) to proxy drug target genes and examined the impact of sildenafil and contraceptives on mental disorders using SMR and IVW‐MR methods.

**Results:**

We found that an increased genetically predicted AFS was associated with decreasing anxiety disorders, bipolar affective disorders, depression, disturbance of activity and attention (DAA), and post‐traumatic stress disorder. An increased genetically predicted NSP was associated with increasing anxiety disorders, bipolar affective disorders, depression, and post‐traumatic stress disorder. SMR analysis found that PDE11A, instead of PDE5A, a drug target for sildenafil, causes DAA. Suggestive evidence was observed regarding the positive association between progesterone receptor expression and risk of depression. Notably, given the exploratory nature of the multiple analyses, these findings require replication in independent cohorts to confirm causal relationships.

## Graphical abstract

Mendelian randomization analysis was conducted to investigate whether sexual behavior, PDE5A/11A, and PGR affect mental disorders. The negative sign (−) indicates a protective effect (reduced risk), whereas the positive sign (+) indicates a risk effect (increased risk). UVMR, Univariable Mendelian Randomization; MVMR, Multivariable Mendelian Randomization; SMR, Summary‐data‐based MR; PDE5A/11A, phosphodiesterase 5A/11A; PGR, progesterone receptor.

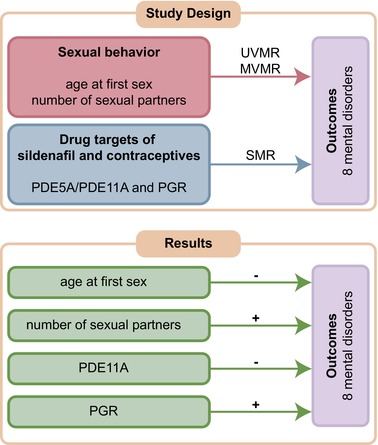

**Abbreviations**
AFS: Age at first sex
AnxD: Anxiety disorders
BD: Bipolar affective disorders
BN: Bulimia nervosa
CI: Confidence intervals
DAA: Disturbance of activity and attention
eQTL: Expression quantitative trait loci
GWAS: Genome‐wide association study
IVs: Instrumental variables
IVW‐MR: Inverse‐variance‐weighted MR
MR: Mendelian randomization
MVMR: Multivariable Mendelian randomization
NHANES: National Health and Nutrition Examination Survey
NSP: Number of sexual partners
OR: Odds ratios
PDE5A/11A: Phosphodiesterase 5A/11A
PGR: Progesterone receptors
PTSD: Post‐traumatic stress disorder
RCT: Randomized controlled trials
SCZ: Schizophrenia
SMR: Summary‐data‐based Mendelian randomization
SNP: Single nucleotide polymorphisms

## 1 Introduction

Mental disorders have become a serious public health challenge. The Global Burden of Disease, Injuries, and Risk Study confirmed mental disorders represented the second leading cause of disability‐related burden [1]. Of these, depression and anxiety are the largest components of the mental disorders [2].

The etiology and pathogenesis of mental disorders are multifactorial, involving genetic and environmental factors [3, 4, 5, 6]. Mental disorders are prevalent among young adults, which may have originated in childhood or adolescence [7]. Specifically, early adolescent problem behaviors are associated with mental disorders [7, 8, 9]. Multiple cross‐sectional studies suggest that early sexual initiation, particularly at a very young age, is associated with a higher incidence of depression, anxiety, self‐injurious behaviors, and suicide attempts among adolescents. A study of 11,406 students (mean age 15.39 ± 0.87 years) revealed that students who had experienced sexual initiation exhibited significantly higher risks for depression (aOR 1.871), anxiety (aOR 2.190), self‐injurious behavior (aOR 2.892), severe suicidal ideation (aOR 2.259), and suicide attempts (aOR 3.091) compared to those who had not. Critically, these adverse associations were substantially stronger among students who had their sexual initiation at or before the age of 15 [10, 11, 12, 13, 14, 15, 16, 17, 18]. Similarly, individuals who had more sexual partners have an increased risk for multiple mental disorders [19]. Additionally, mental disorders exhibited gender differences, which were associated with different sexual behaviors [20]. Females who engaged in sexual intercourse at an earlier age showed more depressive symptoms, and males who had more sexual partners experienced more anxiety symptoms [11, 21].

Sildenafil and hormonal contraception are always mentioned in sexual behavior. Sildenafil is utilized as a phosphodiesterase 5 (PDE5) inhibitor for the treatment of erectile dysfunction, which in turn produces a range of side effects because of cross‐reactivity with PDE6/11 [22]. PDE11A is highly involved in brain function and mental‐related disorders [23]. A deficiency of PDE11A contributes to cognitive, associative social memory, and social approach behavioral impairments [24, 25]. The progestin component of hormonal contraceptives is the main source of contraceptive effectiveness [26]. Use of progestin products can increase the risk of depression, suicide, and suicide attempts, and the relative risk is highest in adolescent females [27, 28].

However, it is difficult to determine the causal effect between sexual behaviors and mental disorders because of the possible confounders (e.g., education and sleeplessness), recall bias, and misreporting of self‐reported sexual behaviors [29, 30]. Randomized controlled trials (RCTs) are considered the “gold standard” for studying causality but require a long time and substantial effort [31]. A method known as Mendelian randomization (MR) tries to overcome these limitations. In MR, phenotypically closely related single nucleotide polymorphisms (SNPs) randomly assigned at conception are utilized to investigate the potential causal relationship between exposures and outcomes. It is possible to consider MR as a natural randomized controlled trial, thereby avoiding the effects of measurement errors, confounders, and reverse causality that are inherent to observational studies [32].

In our study, we carried out a two‐sample univariable Mendelian randomization (UVMR) and multivariable MR (MVMR) [33, 34] to examine the causality between sexual behavior and eight types of mental disorders, including anxiety disorders (AnxD), bipolar affective disorders (BD), bulimia nervosa (BN), disturbance of activity and attention (DAA), post‐traumatic stress disorder (PTSD), schizophrenia (SCZ), anorexia, and depression. Further, we analyze the effect of sex difference on the above causal relationship. The data from the 2015–2016 National Health and Nutritional Examination Surveys (NHANES) were relied upon to make enhancements to the understanding of the relationship between sexual behavior and mental disorders. Lastly, we explored the role of sildenafil's targets, PDE5A/11A and progesterone receptors (PGR), in triggering mental disorders.

## 2 Methods

**FIGURE 1:**
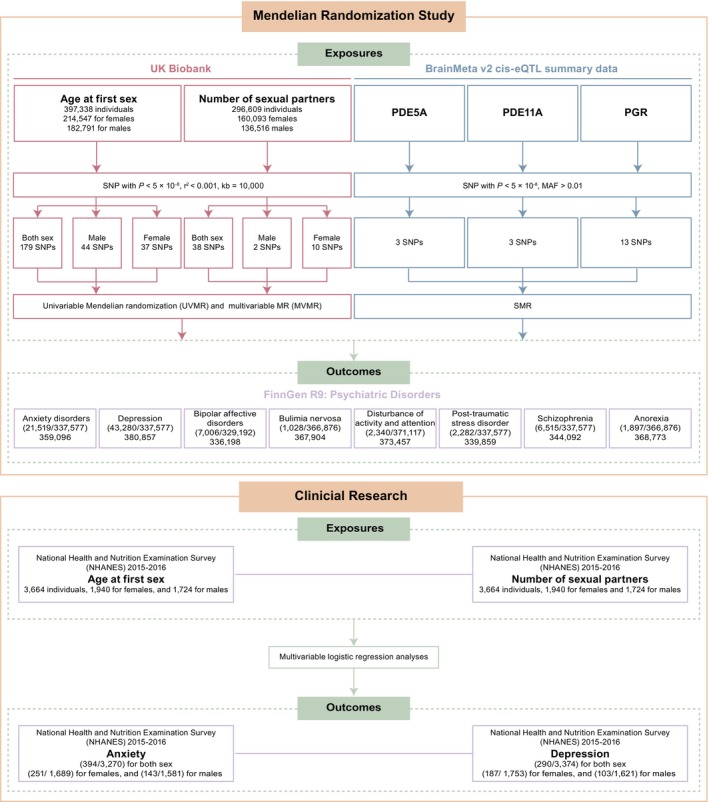
Study overview. Mendelian randomization analysis was conducted to investigate whether sexual behavior, PDE5A/11A, and PGR are associated with mental disorders. A multivariable logistic regression analysis was performed to study the association of sex hormones to anxiety and depression. eQTL, expression quantitative trait loci. PDE5A/11A, phosphodiesterase 5A/11A; PGR, progesterone receptor.SMR, Summary‐data‐based MR; SNP, single nucleotide polymorphism.

Figure 1 depicts a schematic flowchart outlining the analysis performed. On the basis of publicly available summary‐level data from genome‐wide association studies (GWAS), we used two‐sample UVMR to explore the total effect of age at first sex (AFS) (females, males, and both sexes) and the number of sexual partners (NSP) (females, males, and both sexes) on eight types of mental disorders, respectively. Then, we conducted an MVMR design to assess the effects of multiple risk factors jointly to rule out pleiotropy from mediators or confounders and eventually get the direct and independent effect of AFS and NSP on main outcomes. Additionally, we conducted multivariate regression analyses to enhance the assessment of the relationship between sexual behavior and psychiatric disorders according to NHANES 2015–2016. Lastly, combined with GWAS and expression quantitative trait loci (eQTL) studies, we further extrapolated the role of sildenafil and contraceptives in mental disorders through their targets or receptors.

### 2.1 Data Sources

In this study, data on sexual behavior, years of schooling, and sleeplessness were accessed through the IEU Open GWAS project (https://gwas.mrcieu.ac.uk/) and UK Biobank (https://www.ukbiobank.ac.uk/). Additionally, mental disorders data were obtained from the R9 FinnGen study (https://finngen.gitbook.io/documentation/data‐download). AFS summary statistics were drawn from GWAS studies, including a total sample size of 397, 338 individuals, 214,547 for females, and 18,279 for males [35]. Since gender‐stratified SNPs for NSP were only available in the 2018 data from UK Biobank, we used the summary statistics consisting of a total sample size of 296,609 for NSP, 160,093 for males, and 136,516 for females. The sample size of each mental disorder was detailed in Table S1. No overlap of data between exposures and outcomes. Population heterogeneity was avoided by relying solely on the European population's summarized data [36].

NHANES is a national, continuous, cross‐sectional study in the United States. It investigates the health and nutritional status of adults and children in the United States through a multistage probability sampling method [37]. Through selection, we included 3664 individuals in our observational research described in further detail in Tables S2 and S3.

The discovery brain drug‐targeted genes (i.e., PDE5A/11A, and PGR) eQTL dataset was obtained from https://yanglab.westlake.edu.cn/data/brainmeta/cis_eqtl/.

As this study drew upon publicly available summary data, ethics approval was not required.

### 2.2 Selection of Genetic Instruments

Single nucleotide polymorphisms (SNPs) were utilized as instrumental variables (IVs) in the MR study, were associated with the exposure at statistical significance (*p* < 5 × 10^−8^), and were independent after a strict clumping procedure (R^2^ = 0.001, kb = 10,000). For AFS, full 272 SNPs were extracted from the UK biobank [35], but only 179 SNPs were extracted from mental disorders data from the FinnGen biobank. Likewise, we obtained 37 SNPs for female AFS and 44 SNPs for male AFS. In addition, 38 SNPs for NSP, 10 SNPs for female NSP, and 2 SNPs for male NSP were available. Additionally, the dependent effects of each SNP on the mental disorders results are displayed in Tables S4 and S9.

The common (minor allele frequency > 1%) eQTL single SNPs were significantly associated (*p* < 5.0 × 10–8) with PDE5A/11A, and PGR expression in the brain (Table S10). Only cis eQTL were included in this study to generate genetic tools. The F‐statistic is used to estimate the power of the instrumental variables, and it is less likely for a weak instrumental variable if F > 10 [38]. Details of the statistical power calculations for the Mendelian randomization analyses are provided in Table S17.

### 2.3 Mendelian Randomization Estimates

We performed the 2‐sample UVMR and MVMR with the R package. Two‐sample MR in R version 4.2.1. For UVMR, a primary analysis on the basis of random‐effects inverse‐variance weighting (IVW) was performed to estimate the causal association between each sexual behavior and mental disorders [39]. Additionally, we supplemented the weighted median [40], MR Egger [41], simple mode, and weighted mode [42] to ensure the robustness and reliability of the results. In fact, vertical pleiotropy or mediation (the genetic instrument for mental disorders is associated with other traits) is potentially present, and horizontal pleiotropy is common in UVMR. Thus, we performed MVMR to assess confounders, which considered potential genetic overlap directly in the estimation [43, 44, 45, 46, 47].

When employing eQTL as a tool, the summary‐data‐based MR (SMR) method is the primary method [48]. The 2‐sample MR analysis on the basis of SMR software, version 1.03 (https://cnsgenomics.com/software/smr/#Overview).

### 2.4 Sensitivity Analyses

The *p*‐value for MR‐Egger intercept was applied to test for directional horizontal pleiotropy [41], where the genetic variants influence the risk of mental disorders through an alternative pathway. When our results showed significant horizontal pleiotropy, the outliers (*p* < 0.05) were eliminated by MR‐PRESSO, and the remaining SNPs were recalculated in the primary IVW analysis. Cochran's Q statistic was computed to assess the degree of heterogeneity through MR‐Egger estimates. Additionally, the “leave‐one‐out” sensitivity analysis was also conducted to examine the pleiotropy by excluding each SNP in turn. The results were presented more clearly in the forest plots, scatterplots, leave‐one‐out plots, and funnel plots [43]. This study involved a substantial amount of both exposure and outcomes data, and we consistently use conventional *p* < 0.05 to indicate statistical differences. We reported the causal effect between genetically predicted sexual behavior (AFS and NSP) and risk of mental disorders by odds ratios (ORs) with 95% confidence intervals (CIs).

We conducted SMR with heterogeneity in dependent instruments (HEIDI) to test if the resulting association statistics between PDE5A/11A and PGR expression and sexual behaviors were due to the linkage scenario, with *p*‐values < 0.05 suggesting linkage.

## 3 Results

### 3.1 Total Effect of Sexual Behavior on Mental Disorders

**FIGURE 2:**
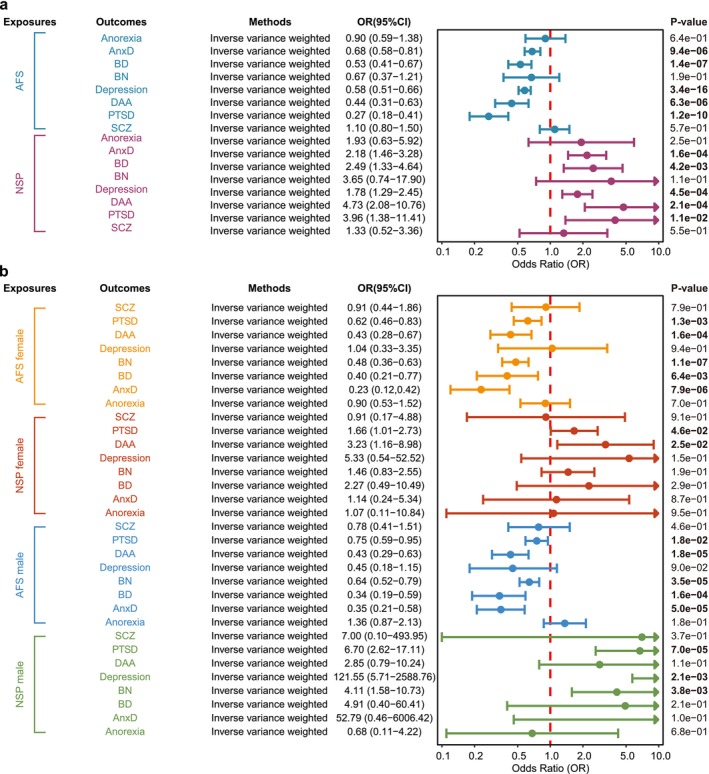
(a) Associations between genetically predicted sexual behavior and mental disorders. (b) Gender differences in sexual behavior and susceptibility to mental disorders. AnxD, anxiety disorders; BD, bipolar affective disorders; BN, bulimia nervosa; CI, confidence interval; DAA, disturbance of activity and attention; OR, odds ratio; PTSD, post‐traumatic stress disorder; SCZ, schizophrenia.

For IVs applied to sexual behavior, the median F‐statistics were 17.37 for AFS and 15.17 for NSP (Table S11), indicating that weak instrument bias is unlikely to exist. In the primary UVMR, the main IVW exhibited a protective effect of later AFS on five mental disorders and an adverse effect of increasing NPS on five mental disorders (Figure 2a). An increased genetically predicted AFS was associated with decreasing DAA, AnxD, BD, depression, and PTSD. Besides, genetically predicted NSP was associated with increasing DAA, AnxD, BD, depression, and PTSD. Both earlier AFS and increasing NPS contributed to DAA, AnxD, BD, depression, and PTSD. However, we failed to find a causal effect of AFS on anorexia, BN, and SCZ, nor did we find causal effects of NSP on these mental disorders (Figure 2a). The results of the sensitivity analysis were generally consistent (Tables S12 and S13, Figure S18). MR–Egger intercepts analysis showed *p* > 0.05 in all results, indicating no horizontal pleiotropy for these risk factors (Table S12). However, several heterogeneity was present (Table S13). It should be emphasized that all the above results are based on exploratory analysis, and the conclusions need to be verified by further confirmatory studies.

### 3.2 Total Effect of Gender‐Specific AFS on Mental Disorders

We performed an exploratory analysis to investigate potential differences between genders. The median F‐statistics of AFS for females and males were 16.53 and 15.40, respectively. The median F‐statistics of NSP for females and males were 14.92 and 12.98, respectively (Table S11). We used genetic instruments to mimic AFS and NSP in females and males separately, accounting for gender differences in mental disorders. Genetically predicted female AFS was associated with DAA, AnxD, BD, depressionA, and PTSD. Similarly, genetically predicted AFS in males was associated with AnxD, BD, depression, DAA, and PTSD. Moreover, genetically predicted NSP in males was associated with an increased risk for AnxD and BD. However, genetically predicted NSP in males was associated with AnxD, BN, and depression (Figure 2b). Sensitivity analyses displayed generally consistent results (Tables S12 and S13; Figures S9 and S23). We did not detect horizontal pleiotropy, but several heterogeneities in the estimates for female NSP, AFS, and male AFS (Tables S12 and S13). Since there were insufficient numbers of male SNPs, MR‐Egger Intercept and Cochran's Q statistic were not performed in male NSPs.

### 3.3 Direct Effects of Sexual Behavior on Mental Disorders

Given the identified effects of genetically predicted sleeplessness, years of schooling, and other sexual behaviors on anxiety and depression, we added these three risk factors for mental disorders to the MVMR model. This exploratory analysis was conducted to estimate the direct effects of AFS and NSP on eight mental disorders.

**FIGURE 3:**
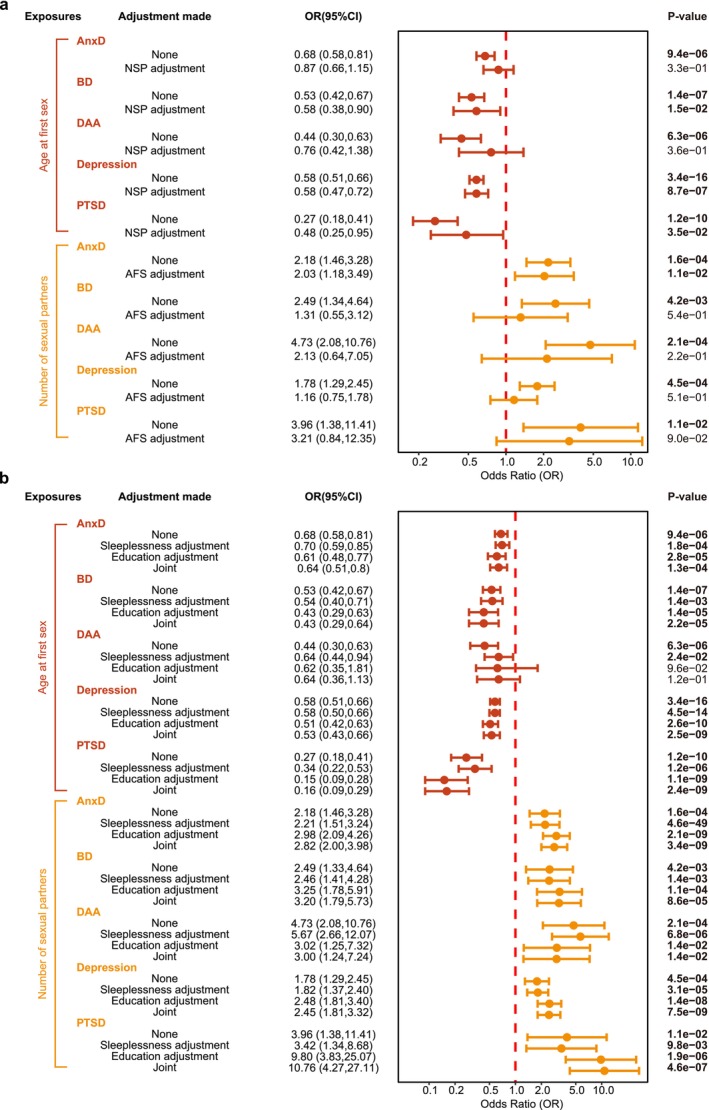
The relationship between sexual behaviors and depression or AnxD in Multivariable Mendelian randomization. (a) Direct effects of sexual behavior on mental disorders, accounting for other sexual behaviors. (b) Direct effects of sexual behavior on mental disorders, accounting for education and/or sleeplessness.

Genetically predicted AFS still had a protective effect on BD, depression, and PTSD. NSP only had an adverse effect on AnxD after mutual adjustment for the other sexual behaviors. However, the AFS effect on AnxDand DAA diminished after adjusting for NSP. Similarly, the effect of NSP on BD, DAA, depression, and PTSD decreased after adjusting for AFS (Figure 3a; Table S15).

The median F‐statistics of the MVMR model were detailed in Table S14.

The effects of sexual behavior on all mental disorders except DAA were maintained and even significantly strengthened by adding education and/or sleeplessness to the MVMR (Figure 3b; Table S15).

### 3.4 Observational Results of AnxD and Depression on Sexual Behavior in NHANES

To provide external validation for the aforementioned exploratory findings, we further incorporated data from the NHANES observational study for analysis. Table S3 illustrates the baseline characteristics of participants by depression and anxiety categories. There were statistically significant differences in sex, education, sleep duration, AFS and NSP between the No and Yes depression and anxiety group, with the exception of education between anxiety group.

The results of multivariable logistic regression analyses of AFS and NSP in relation to depression and anxiety are displayed in Figure S24. There was a significant association between AFS and two mental disorders in the non‐adjusted model and the model (adjusted for NSP). The association between NSP and two types of mental disorders remains significant in both non‐adjusted models and the model (adjusted for AFS).

In Model 1 (adjusted for sleep disorders), Model 2 (adjusted for education), Model 3 (adjusted for sleep disorders and education), there was a significant inverse association between AFS and two mental diseases, whereas NSP had a significant positive correlation with both mental disorders (Figure 24; Table S16).

### 3.5 PDE5A/11A and PGR on Mental Disorders

**FIGURE 4:**
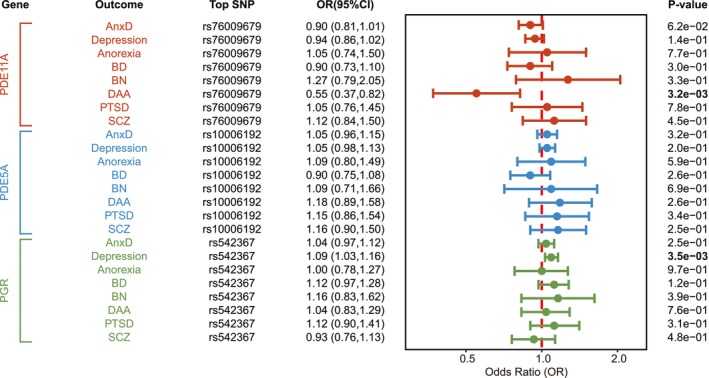
Two‐sample MR results of drug‐targeted genes PDE5A/11A and PGR on mental disorders.

In Figure 4, our exploratory SMR analysis revealed suggestive evidence for the association of the increased expression of the PDE11A gene in the brain (equivalent to a one standard deviation increase) with the lower risk of DAA susceptibility, implying that PDE11A inhibitors might increase the risk of DAA. Suggestive evidence was observed regarding the positive association between PGR expression and risk of depression (Figure 4). No significant association was found between the expression of PDE5A and mental disorders. The resulting association statistics were not a consequence of linkage (*p* > 0.05) in HEIDI test (Table S10).

## 4 Discussion

In the present study, we take genetic information as a premise to assess the possible causal relationship between the two sexual behaviors and mental disorders. We use GWAS summary data by a two‐sample MR analysis to assess the total and direct effect of genetically predicted sexual behavior (AFS and NSP) on mental disorder risk and a wide range of mental disorder outcomes. It was consistent with the observations of AFS and NSP effects on AnxD and depression in NHANES 2015–2016. Also, two‐sample MR combining the GWAS and eQTL datasets showed that the non‐therapeutic target of sildenafil, PDE11A, causes DAA, as well as the hormonal contraceptive's receptor, PGR, which has a role in depression.

UVMR results showed that genetically predicted earlier AFS and increased NSP were positive with mental disorders. Both AFS and NSP played key roles in the development of DAA, AnxD, BD, depression and PTSD. Our results support previous population‐based observational studies, which show that earlier sexual behavior and more sexual partners have negative effects on mental disorders [13, 17, 19]. In our NHANES‐based observational results, the effects were consistent of AFS and NSP on AnxD and depression remained. Reproductive behaviors including AFS had genetic correlations with attention deficit, hyperactivity disorder, major depressive disorder, SCZ, anorexia, and BD [49]. Similarly, previous studies suggest that AFS was associated with anxiety‐related disorders [50]. Nonetheless, when AFS and NSP were added to the MVMR, the causal relationship between AFS and AnxD/DAA disappeared, indicating that earlier AFS that was not accompanied by more sexual partners may not lead to AnxD/DAA. Furthermore, our findings showed a robust causal association of AFS in relation to BD, PTSD, and depression. Previous MR studies have highlighted a causal relationship between NSP and attention deficit and hyperactivity disorder and major depressive disorder [49]. Our MR complemented the positive correlation of NSP with AnxD/BD/PTSD. Additionally, the NSP effect on BD/DAA/depression/PTSD diminished after accounting for AFS. This meant that more NSP without earlier AFS may not cause BD/DAA/depression/PTSD.

The occurrence and development of mental health are related to genetic, environmental, and social factors, among which education is considered to be a protective factor of mental health [51], but sleeplessness is considered to be a negative factor [29]. Higher education may be related to having multiple partners and having an earlier age at sexual debut [52]. Despite the joint model combining any education and/or sleeplessness, MVMR demonstrated the robustness of sexual behavior to AnxD and depression outcomes, which was consistent with the results of the multi‐logistic model analysis in NHANES 2015–2016.

Moreover, the effects of sexual behaviors on mental disorders have been shown to be sex‐specific.

There was a greater effect of female AFS on AnxD/depression/PTSD compared to that of male AFS. A growing body of observational literature showed a stronger relationship between early sexual behavior and mental disorders in girls than in boys [10, 17]. It was common for women to report being forced to have sex for the first time, especially among those who had sex before the age of 14 [53]. Sexual abuse in childhood and adolescence often has adverse psychological consequences [54]. The majority of women who had sex before the age of 16 reported regretful and negative feelings [53]. The first involuntary sexual intercourse of females may be the explanation why early AFS of females was more vulnerable to mental disorders. It should be noted that the sex‐specific analyses in the present study are exploratory, aiming to initially uncover potential gender difference trends. The conclusions drawn herein require further validation through cohort studies with larger sample sizes, multi‐center replication, and more in‐depth mechanistic investigations to enhance the reliability and generalizability of the findings. Progesterone is widely used in sexual behavior as one of the important contraceptives, and progesterone acts through PGR receptors. When PGR was identified for the development of mental disorders, we found a role for PGR in depression. Observational studies pointed to this interaction being more sensitive in adolescent girls [28]. This may be one of the important reasons why female risky sexual behavior was strongly associated with depression. Besides, males with NSP were more prone to AnxD/depression/BN, and NSP had robust results with AnxD in MVMR. Previous studies consistently suggest that males who have had more sexual partners experience more anxiety symptoms [21]. Interestingly, in the drug‐targeting gene MR analysis, PDE11A inhibitors but not PDE5A inhibitors were able to cause DAA. DAA prominently features impairments in academic and social functioning [55]. Also, PDE11A was highly expressed in the hippocampus and regulates social behavior through the oxytocin pathway and membrane signaling [25]. In conclusion, PDE11A deficiency was probably one of the pathogenic mechanisms of DAA.

To effectively prevent and manage reproductive health issues and mental disorders, the following key interventions are recommended: systematically advancing comprehensive sexuality education for children and adolescents, delaying the age of first sexual intercourse through scientifically guided reduction of early sexual behavior risks, and providing professional psychological support for adolescents who have engaged in early sexual activity. Empirical evidence indicates that school‐based comprehensive sexuality education programs significantly delay the onset of first sexual intercourse among adolescents [56]. Concurrently, parallel development of out‐of‐school comprehensive sexuality education systems is essential to provide comprehensive guidance on physical development, health management, and sexual behavior knowledge for adolescents lacking access to formal education channels [57].

## 5 Implications

Mental disorders, especially anxiety and depression, are the leading causes of disability worldwide, and their prevalence is increasing every year. In addition, both reproductive and mental disorders are critical concerns for adolescent well‐being, but little is known about their interactions. Our findings showed that sexual behavior had a broad impact on the development of mental disorders. In addition to its clinical implications, our study has important public health implications as well. There have been reports that more than one‐third of American students have had early sexual intercourse, and they are more likely to experience the majority of sexual risk behaviors, including having more sexual partners [58]. Comprehensive sex education is associated with a later age at first sex [59]. We recommend more proactive preventive measures to improve sexual behaviors and promote reproductive health and mental health.

## 6 Strengths and Limitations

This study consists of a few advantages. First, the overriding advantage was the MR study, which avoided possible residual confounders, reverse causality, and recall bias. It is also less time‐consuming, less costly, and more accessible than randomized controlled trials. Two‐sample MR minimizes the ‘winner's curse’, that is, the true causal effect is biased towards the observations in one‐sample MR [60]. Second, we used MR methods (UVMR, MVMR) consecutively to thoroughly explore the effect of sexual behavior (AFS and NSP) on mental disorder risk factors and a wide range of mental disorder outcomes, which ensured the reliability of the results. Third, for MR analysis, we stratified exposure by sex in order to determine whether sexual behavior is associated with mental disorders along with gender differences. Finally, only the European population was studied to minimize population structure bias.

Inevitably, there are also several limitations to our study. We used only European population‐summarized data in our study, which limited the generalizability of our results. Furthermore, we used genetic instruments to mimic female and male sexual behavior separately; however, given the absence of gender‐specific GWAS summary statistics for mental disorders, sex‐specific two‐sample MR analyses could not be performed. In addition, because of the lack of gender‐stratified SNPs in the largest and most up‐to‐date GWAS data on NSP, we obtained data from the UK Biobank for 2018, which resulted in a dramatic reduction in sample size. Lastly, the generalizability of outcomes was absent in the observational study analyses, as the only datasets for depression and anxiety were accessible. For analyses of certain mental disorders, the statistical power was relatively low. This is primarily due to the limited sample size of the GWAS for these specific traits, which restricts the proportion of variance explained by the genetic instruments. Critically, our study involved multiple statistical tests across a wide range of exposures and outcomes; however, we did not apply systematic correction for multiple testing. Coupled with the relatively limited statistical power of some analyses, findings from underpowered analyses should be interpreted as preliminary and indicative. Non‐significant associations in these analyses cannot definitively rule out potential causal relationships, whereas significant associations may suggest relatively strong effects. Future investigations incorporating more diverse and larger‐scale GWAS data will be essential to verify the robustness of these findings.

In conclusion, the current study has focused on the causal role of sexual behavior on mental disorders, but the underlying mechanisms have yet to be unraveled.

## 7 Conclusion

To conclude, combining MR methods with a representative observational study in NHANES, this study showed evidence that earlier AFS and more NSP were related to an increased risk of suffering from a variety of mental disorders. Preventing these risk factors can be effective in promoting reproductive and mental health.

## Author Contributions

K.Z., Z.L., and Q.L. designed the study. Z.L. obtained data and performed data analyses. K.Z. and Q.L. wrote the manuscript. Z.J., R.H., H.W., J.Y., and X.L. interpreted the results. J.S., H.Z., and H.H. contributed to the discussion and edited the manuscript. H.H. is the guarantor of this work and, as such, had full access to all data in the study and takes responsibility for the integrity and accuracy of data analysis. All authors critically revised the manuscript and approved it in its final form.

## Funding

National Key Research and Development Program of China (Nos. 2022YFC2703000 and 2021YFC2700603), The National Natural Science Foundation of China (Nos. 82088102, 82171613, and 82001645), Collaborative Innovation Program of Shanghai Municipal Health Commission (No. 2020CXJQ01), Shanghai Frontiers Science Center of Reproduction and Development, CAMS Innovation Fund for Medical Sciences (No. 2019‐I2M‐5‐064), Clinical Research Plan of SHDC (No. SHDC2020CR1008A), Shanghai Clinical Research Center for Gynecological Diseases (22MC1940200), Shanghai Urogenital System Diseases Research Center (2022ZZ01012), Shanghai Frontiers Science Research Center of Reproduction and Development, and Key Discipline Construction Project (2023–2025) of Three‐Year Initiative Plan for Strengthening Public Health System Construction in Shanghai (GWVI‐11.1‐35).

## Ethics Statement

The authors have nothing to report.

## Conflicts of Interest

The authors declare no conflicts of interest.

## Supporting information


**Table S1:** Overview of exposure and 8 outcomes GWAS.
**Table S2:** Assessment of exposure and outcome symptoms.
**Table S3:** The baseline characteristics of participants in NHANES 2015–2016.
**Table S4:** The genetic associations of AFS on nine mental disorders.
**Table S5:** The genetic associations of NSP on nine mental disorders.
**Table S6:** The genetic associations of female AFS on nine mental disorders.
**Table S7:** The genetic associations of male AFS on nine mental disorders.
**Table S8:** The genetic associations of female NSP on nine mental disorders.
**Table S9:** The genetic associations of male NSP on nine mental disorders.
**Table S10:** Drug‐Target Mendelian randomization analyze.
**Table S11:** Different statistical methods for MR analysis for genetic associations between exposures and mental disorders in univariable MR.
**Table S12:** Summary for directional horizontal pleiotropy tests in univariable MR.
**Table S13:** Cochran's Q statistic as a measure of heterogeneity between individual SNP‐effects in univariable MR.
**Table S14:** F‐statistic in multivariate Mendelian randomization analyze.
**Table S15:** Full result of multivariate MR estimates for the association between sexual behavior and mental disorders.
**Table S16:** The results of multivariable logistic regression analyses.
**Table S17:** Statistical power in univariable Mendelian randomization analyses.

**Figure S1:** Scatter plot of AFS on mental disorders.
**Figure S2:** Funnel plot of AFS on mental disorders.
**Figure S3:** Leave‐one‐out plot of AFS on mental disorders.
**Figure S4:** Forest plot of AFS on mental disorders.
**Figure S5:** Scatter plot of NSP on mental disorders.
**Figure S6:** Funnel plot of NSP on mental disorders.
**Figure S7:** Leave‐one‐out plot of NSP on mental disorders.
**Figure S8:** Forest plot of NSP on mental disorders.
**Figure S9:** Scatter plot of female AFS on mental disorders.
**Figure S10:** Funnel plot of female AFS on mental disorders.
**Figure S11:** Leave‐one‐out plot of female AFS on mental disorders.
**Figure S12:** Forest plot of female AFS on mental disorders.
**Figure S13:** Scatter plot of male AFS on mental disorders.
**Figure S14:** Funnel plot of male AFS on mental disorders.
**Figure S15:** Leave‐one‐out plot of male AFS on mental disorders.
**Figure S16:** Forest plot of male AFS on mental disorders.
**Figure S17:** Scatter plot of female NSP on mental disorders.
**Figure S18:** Funnel plot of female NSP on mental disorders.
**Figure S19:** Leave‐one‐out plot of female NSP on mental disorders.
**Figure S20:** Forest plot of female NSP on mental disorders.
**Figure S21:** Scatter plot of male NSP on mental disorders.
**Figure S22:** Funnel plot of male NSP on mental disorders.
**Figure S23:** Forest plot of male NSP on mental disorders.
**Figure S24:** The results of multivariable logistic regression analyses in NHANES.

## Data Availability

This study bases on publicly available summary data. The datasets in Mendelian randomization are accessed through the IEU Open GWAS project (https://gwas.mrcieu.ac.uk/), UK Biobank (https://www.ukbiobank.ac.uk/), and R9 FinnGen study (https://finngen.gitbook.io/documentation/data‐download). The analysis of the summary‐based Mendelian randomization method is based on summary‐based Mendelian randomization software, version 1.03 (https://cnsgenomics.com/software/smr/#Overview). Individual‐level data are available via application to the National Health and Nutrition Examination Survey (NHANES) 2015‐2016 (https://wwwn.cdc.gov/Nchs/Nhanes/continuousnhanes/default.aspx?BeginYear=2015).
