# Supplementary material for: Genetic Association of Sexual Behavior, Sildenafil, and Hormonal Contraceptives With Mental Disorders
Source: CNS Neurosci Ther. 2026 Apr 16;32(4):e70861. doi: 10.1002/cns.70861 (PMC13087095; doi:10.1002/cns.70861)

Supplemental Material

| **Figures** |  |
| --- | --- |
| Figure S1. Scatter plot of AFS on mental disorders | Page 2 |
| Figure S2. Funnel plot of AFS on mental disorders | Page 3 |
| Figure S3. Leave-one-out plot of AFS on mental disorders | Page 4 |
| Figure S4. Forest plot of AFS on mental disorders | Page 5 |
| Figure S5. Scatter plot of NSP on mental disorders | Page 6 |
| Figure S6. Funnel plot of NSP on mental disorders | Page 7 |
| Figure S7. Leave-one-out plot of NSP on mental disorders | Page 8 |
| Figure S8. Forest plot of NSP on mental disorders | Page 9 |
| Figure S9. Scatter plot of female AFS on mental disorders | Page 10 |
| Figure S10. Funnel plot of female AFS on mental disorders | Page 11 |
| Figure S11. Leave-one-out plot of female AFS on mental disorders | Page 12 |
| Figure S12. Forest plot of female AFS on mental disorders | Page 13 |
| Figure S13. Scatter plot of male AFS on mental disorders | Page 15 |
| Figure S14. Funnel plot of male AFS on mental disorders | Page 15 |
| Figure S15. Leave-one-out plot of male AFS on mental disorders | Page 16 |
| Figure S16. Forest plot of male AFS on mental disorders | Page 17 |
| Figure S17. Scatter plot of female NSP on mental disorders | Page 18 |
| Figure S18. Funnel plot of female NSP on mental disorders | Page 19 |
| Figure S19. Leave-one-out plot of female NSP on mental disorders | Page 20 |
| Figure S20. Forest plot of female NSP on mental disorders | Page 21 |
| Figure S21. Scatter plot of male NSP on mental disorders | Page 22 |
| Figure S22. Funnel plot of male NSP on mental disorders  Figure S23. Forest plot of male NSP on mental disorders | Page 23  Page 24 |
| Legends for the above figures  Figure S24. The results of multivariable logistic regression analyses in NHANES | Page 25  Page 26 |

| Figure S1. Scatter plot of AFS on mental disorders  **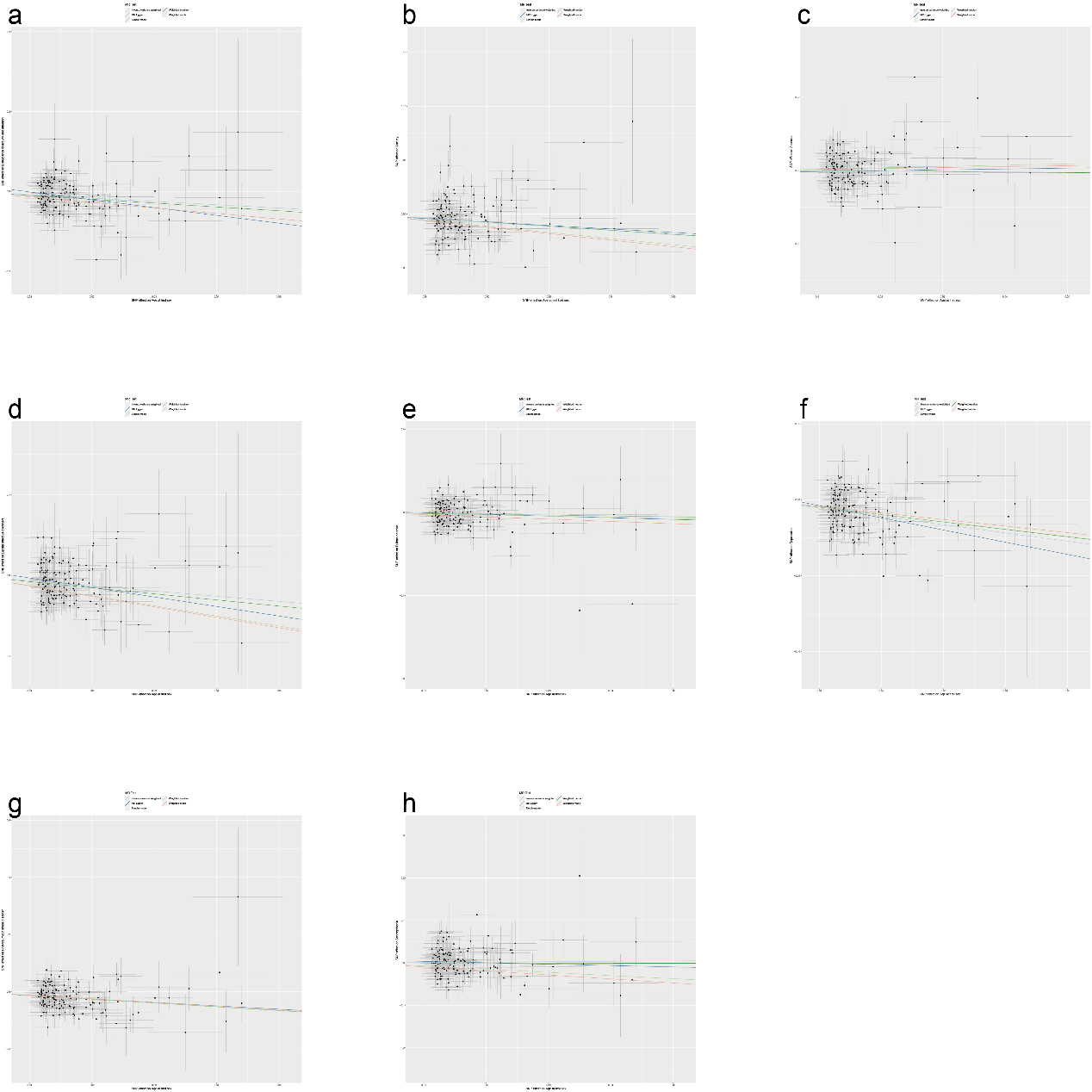** |
| --- |
| Figure S2. Funnel plot of AFS on mental disorders  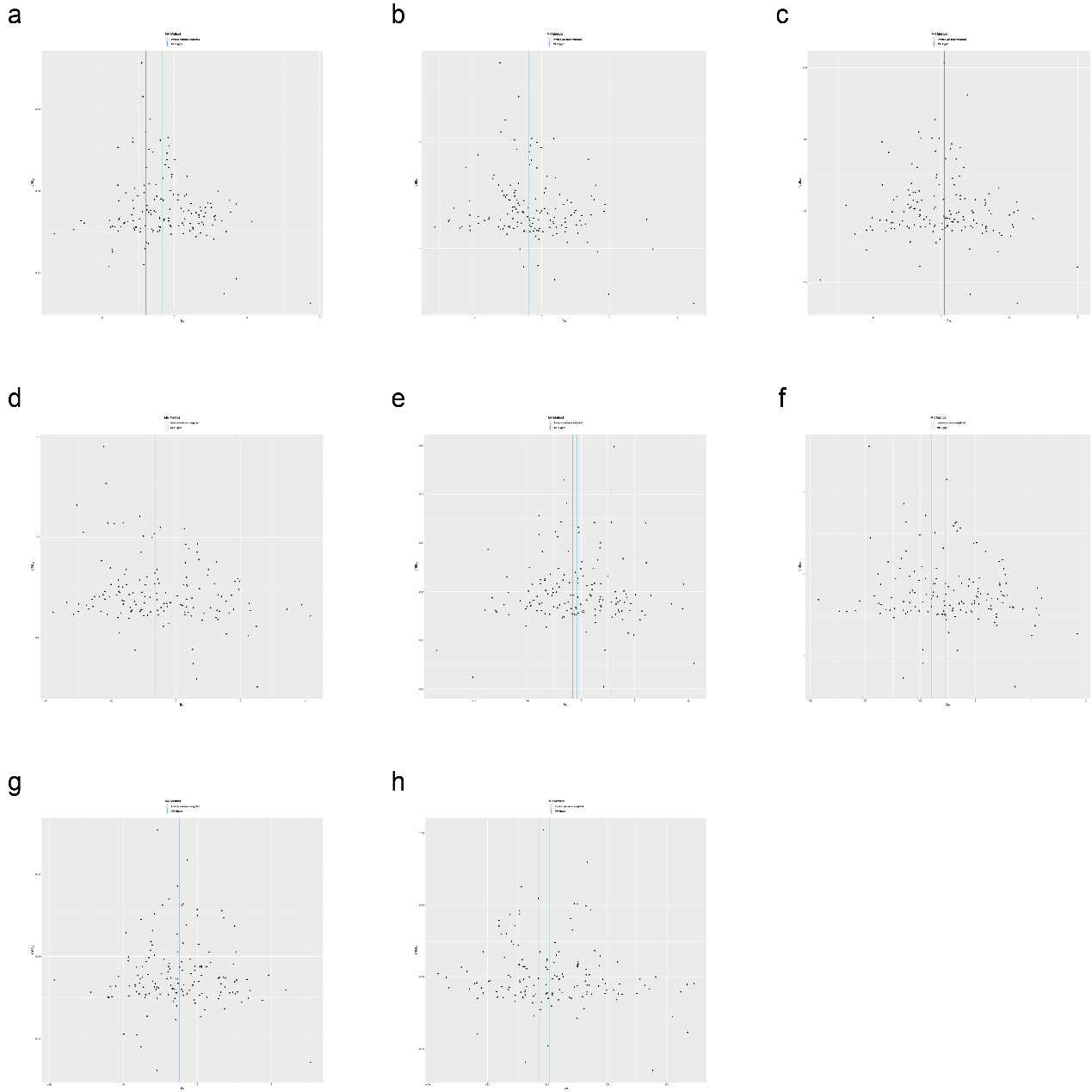 |
| Figure S3. Leave-one-out plot of AFS on mental disorders  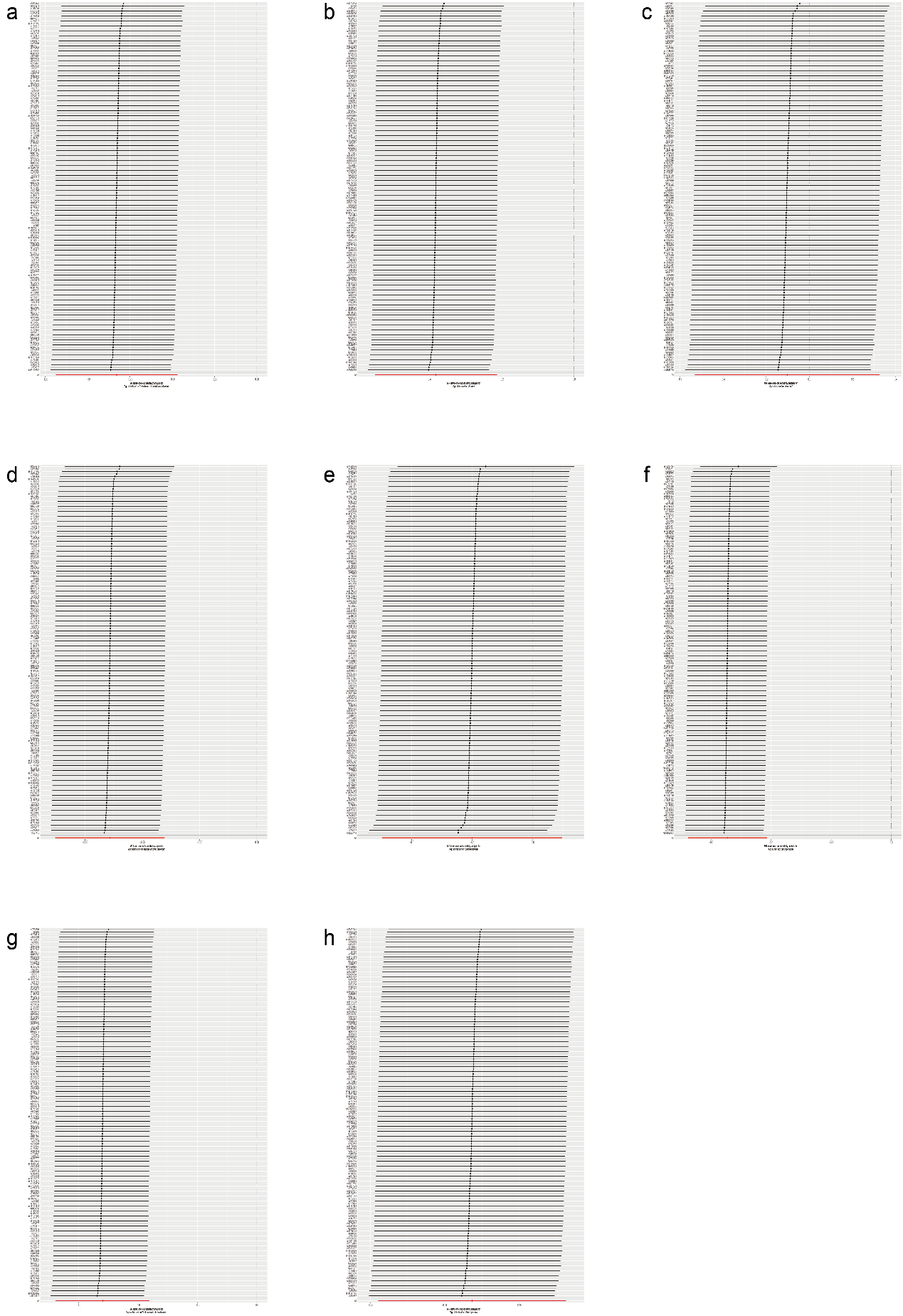 |
| Figure S4. Forest plot of AFS on mental disorders  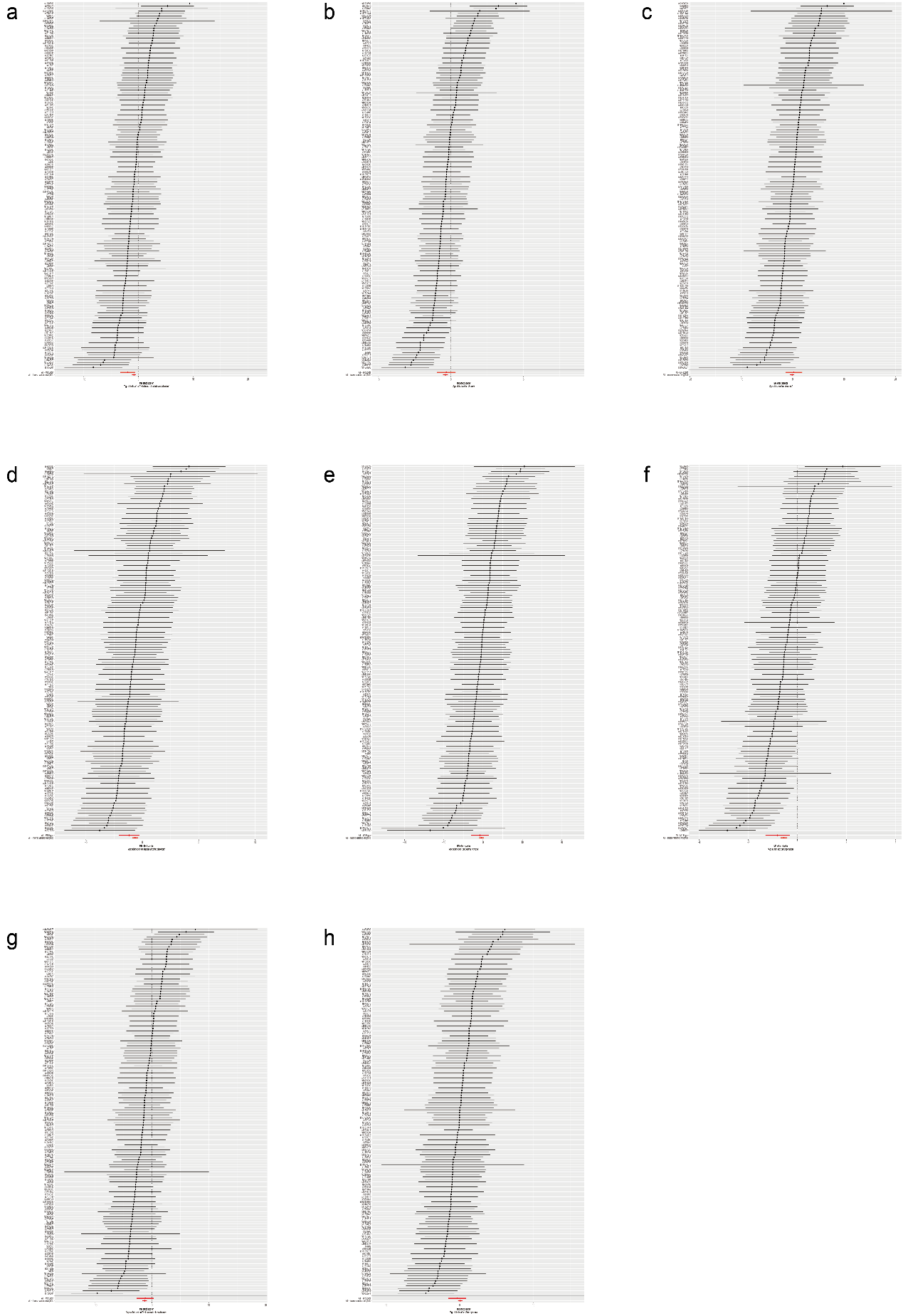 |
| Figure S5. Scatter plot of NSP on mental disorders  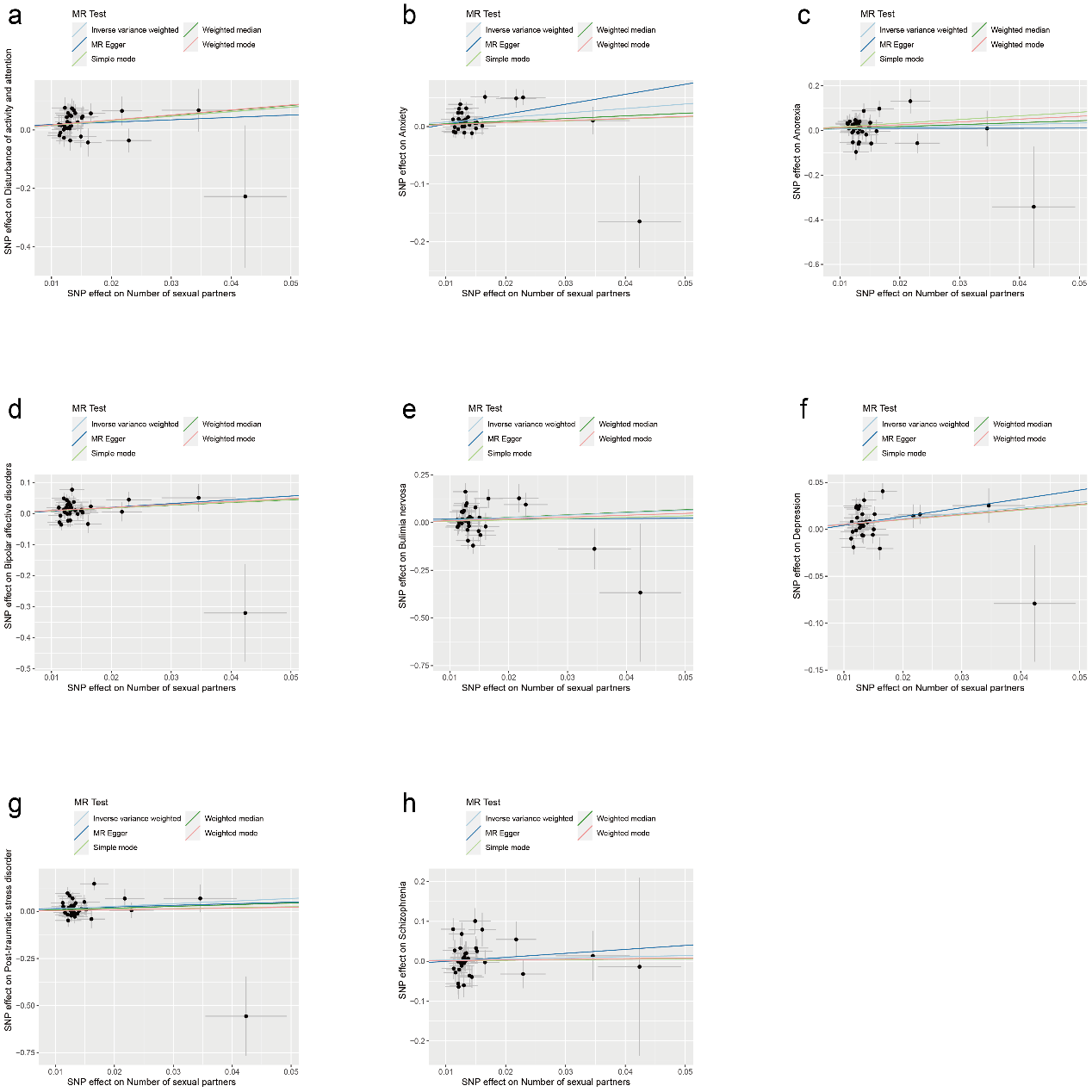 |
| Figure S6. Funnel plot of NSP on mental disorders  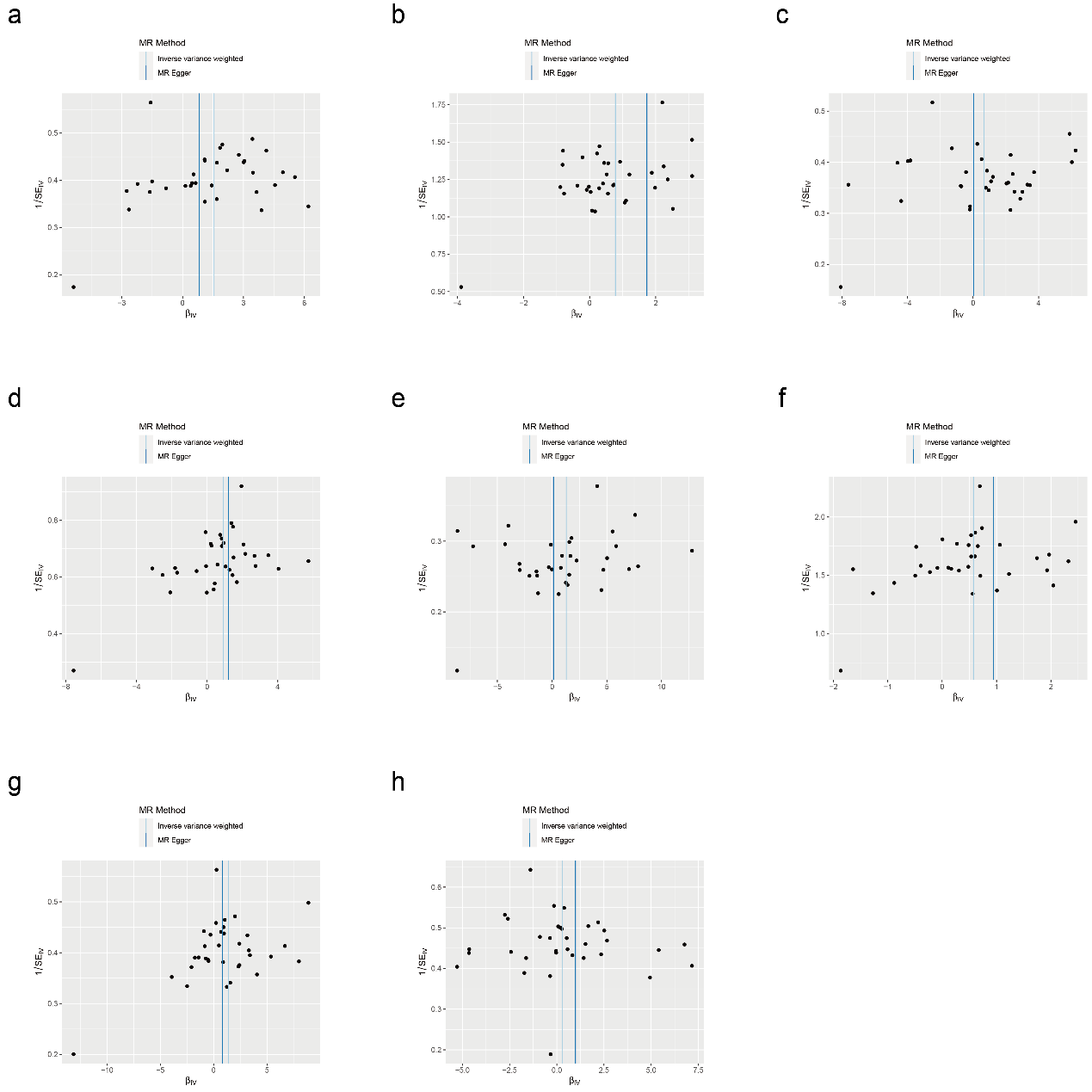 |
| Figure S7. Leave-one-out plot of NSP on mental disorders  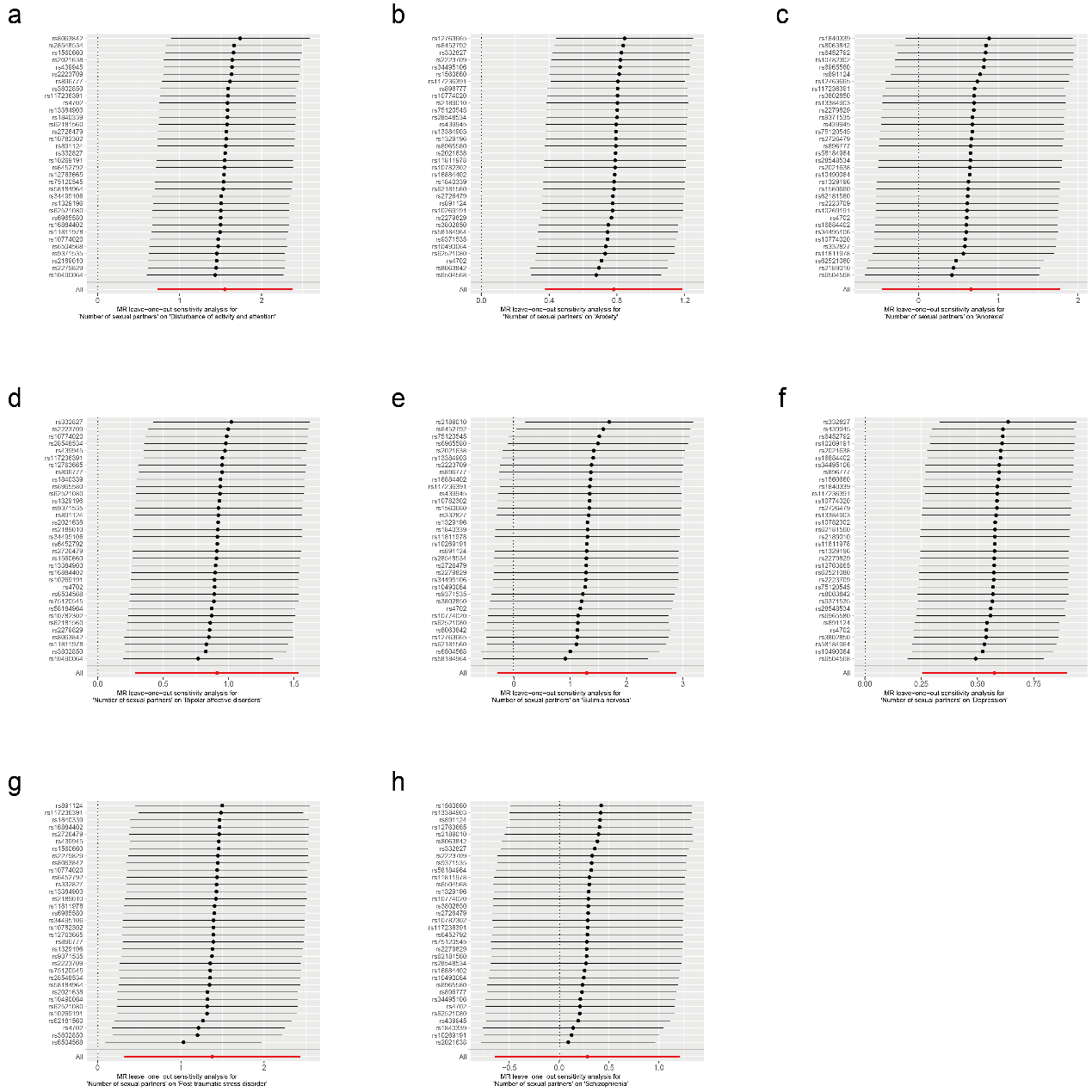 |
| Figure S8. Forest plot of NSP on mental disorders  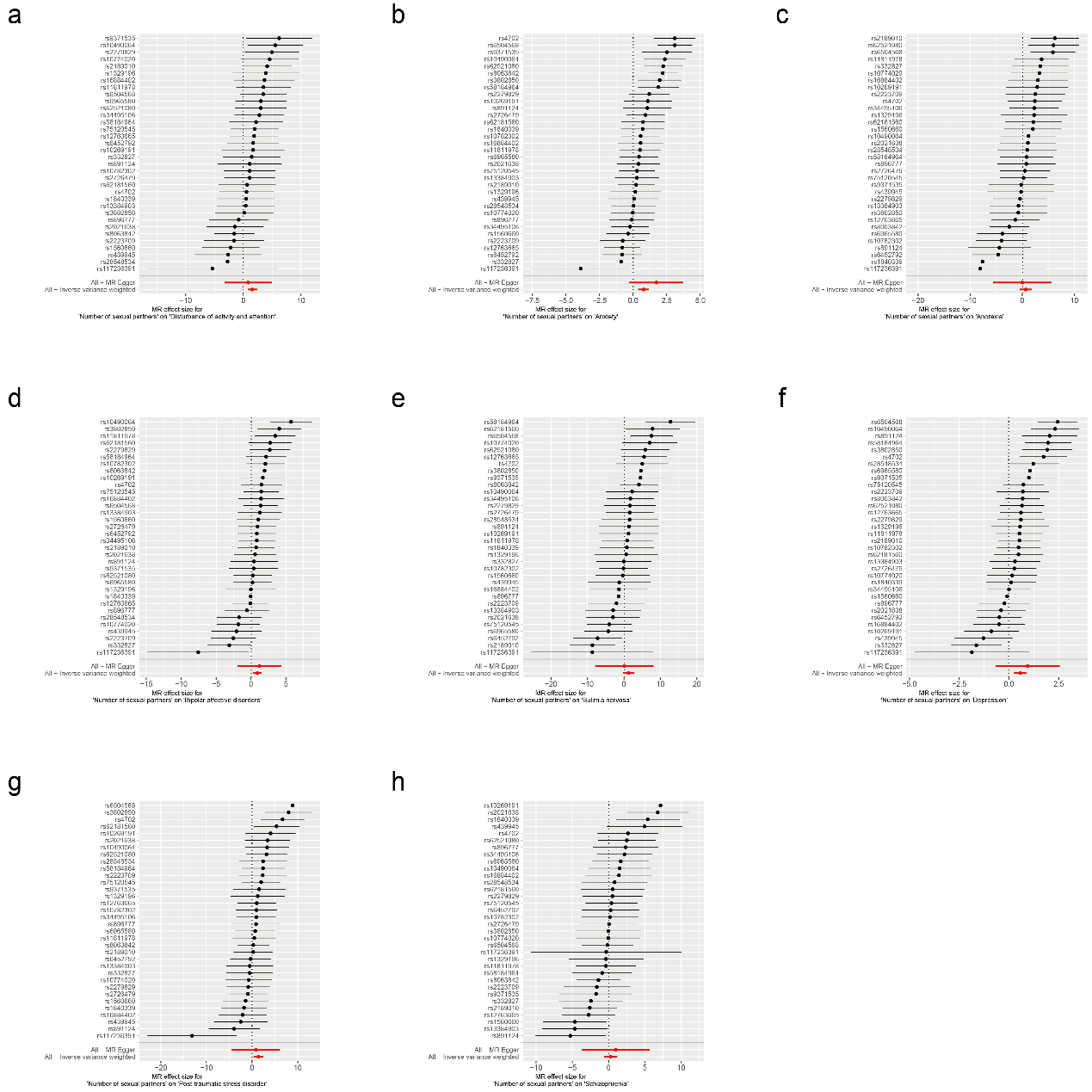 |
| Figure S9. Scatter plot of female AFS on mental disorders  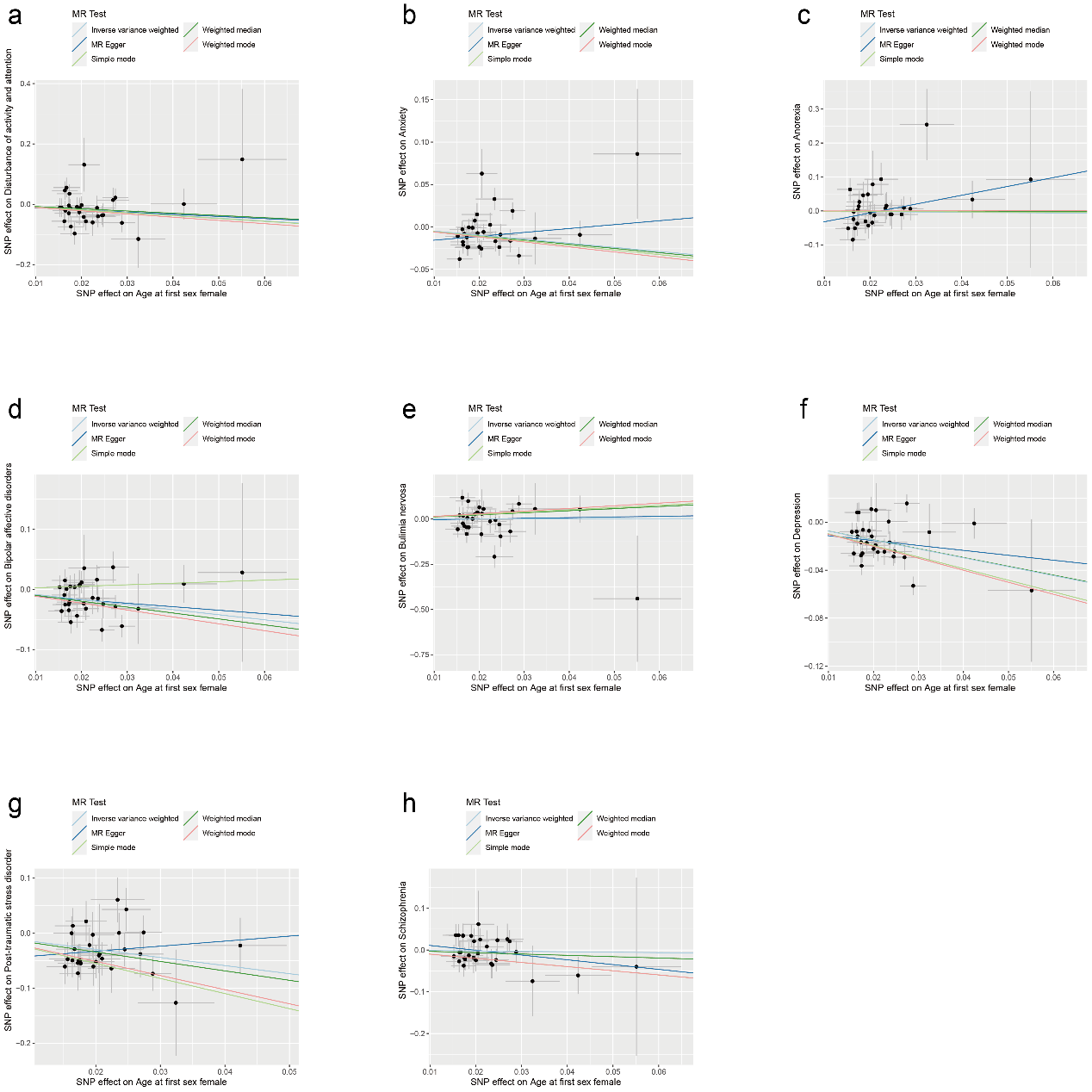 |
| Figure S10. Funnel plot of female AFS on mental disorders  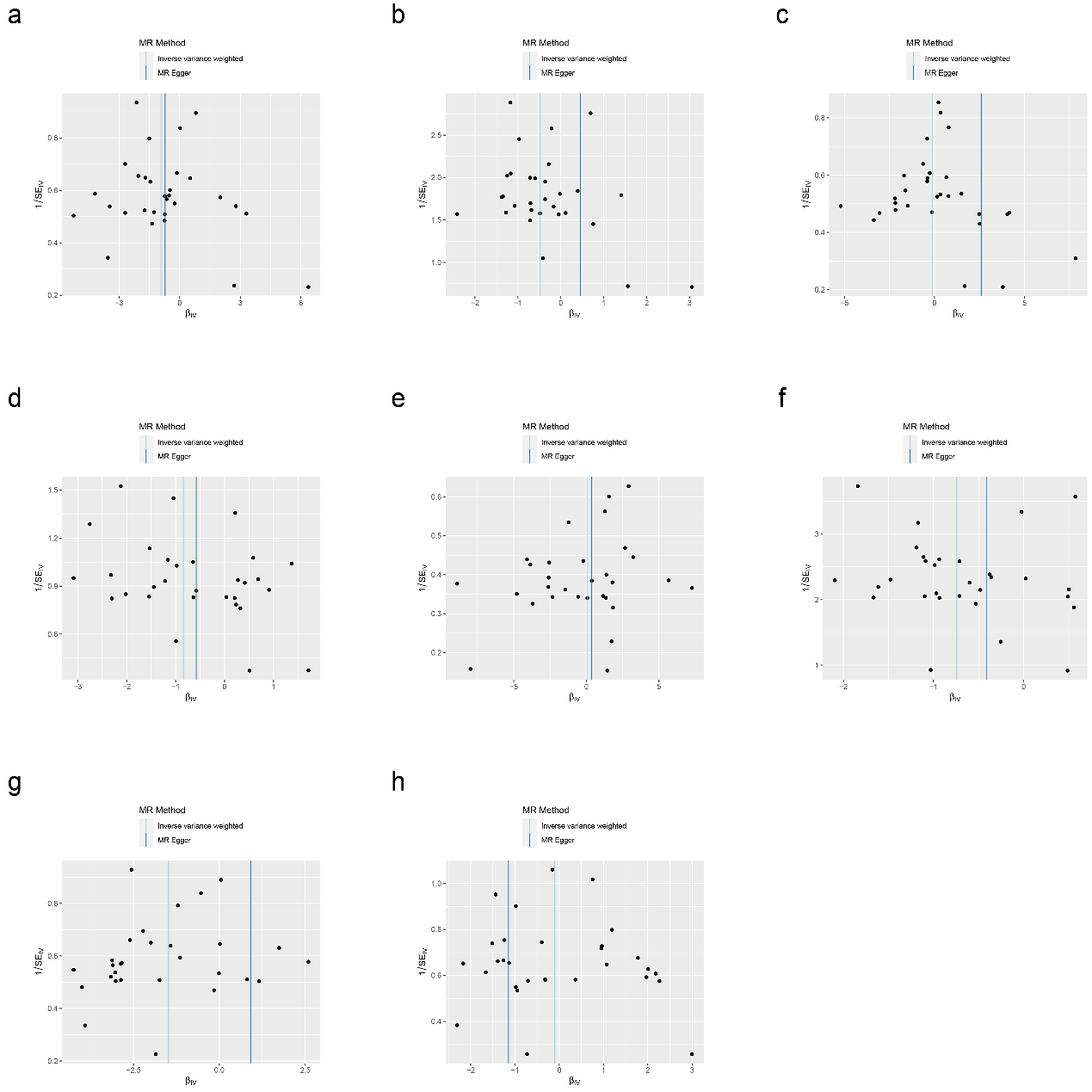 |
| Figure S11. Leave-one-out plot of female AFS on mental disorders  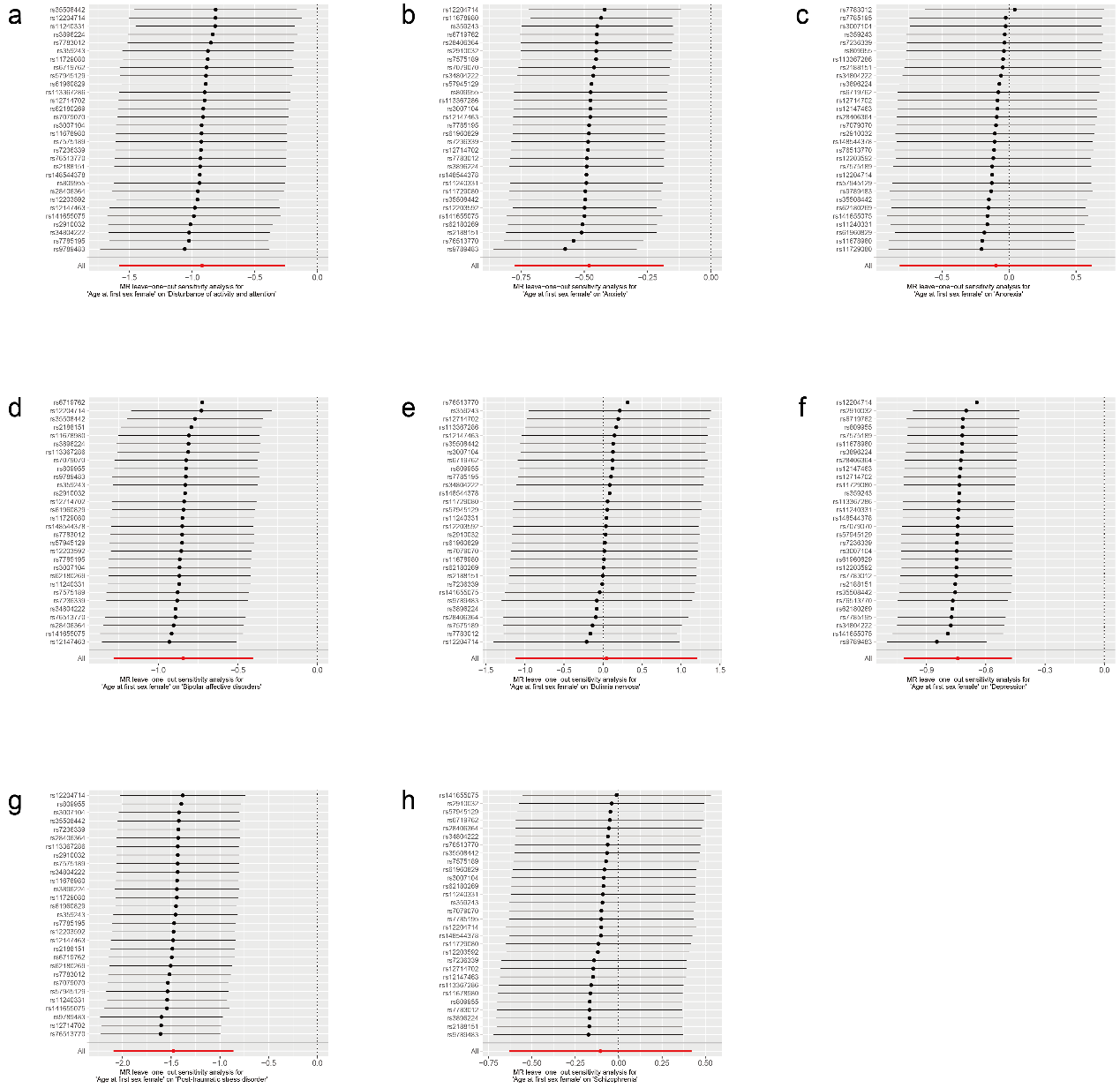 |
| Figure S12. Forest plot of female AFS on mental disorders  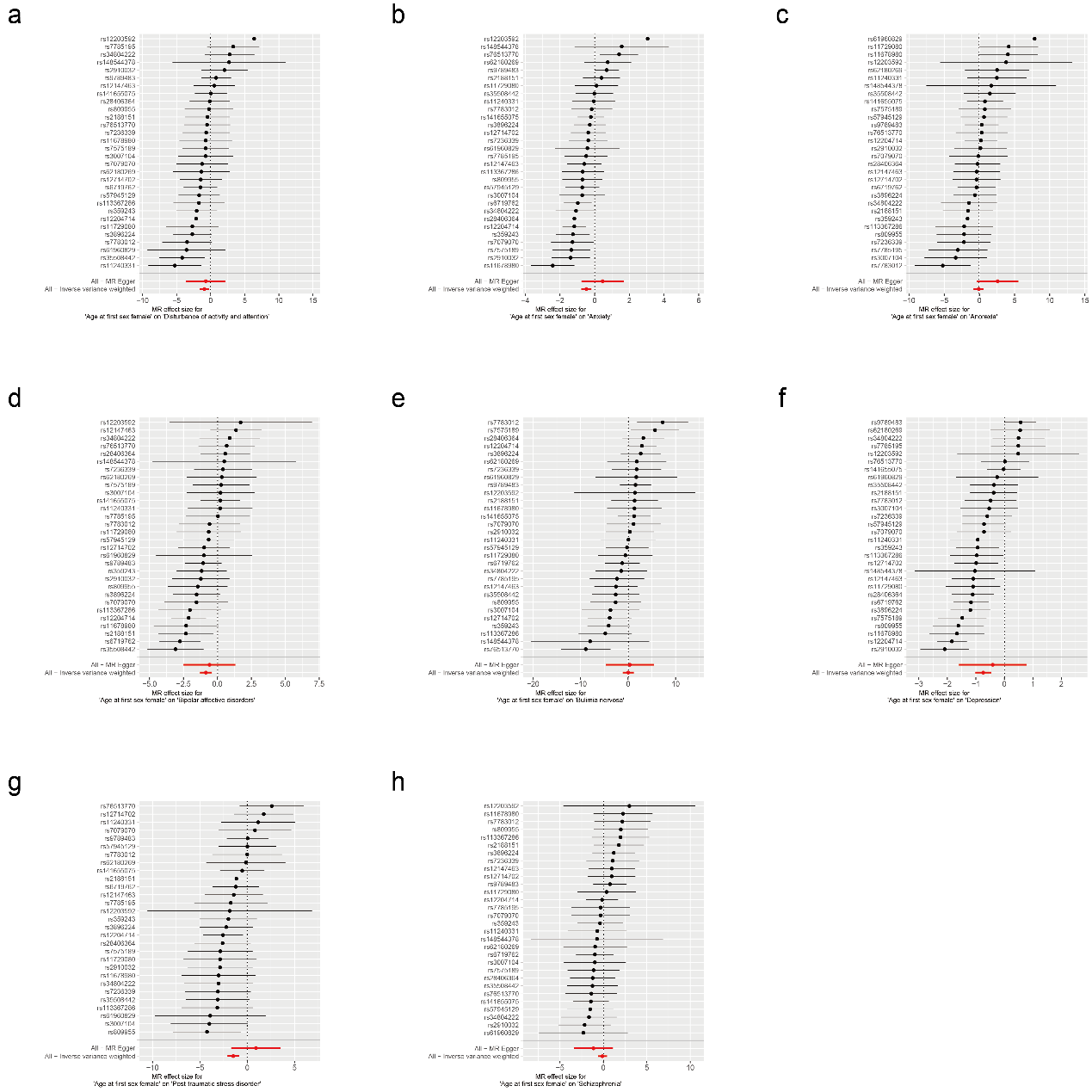 |
| Figure S13. Scatter plot of male AFS on mental disorders  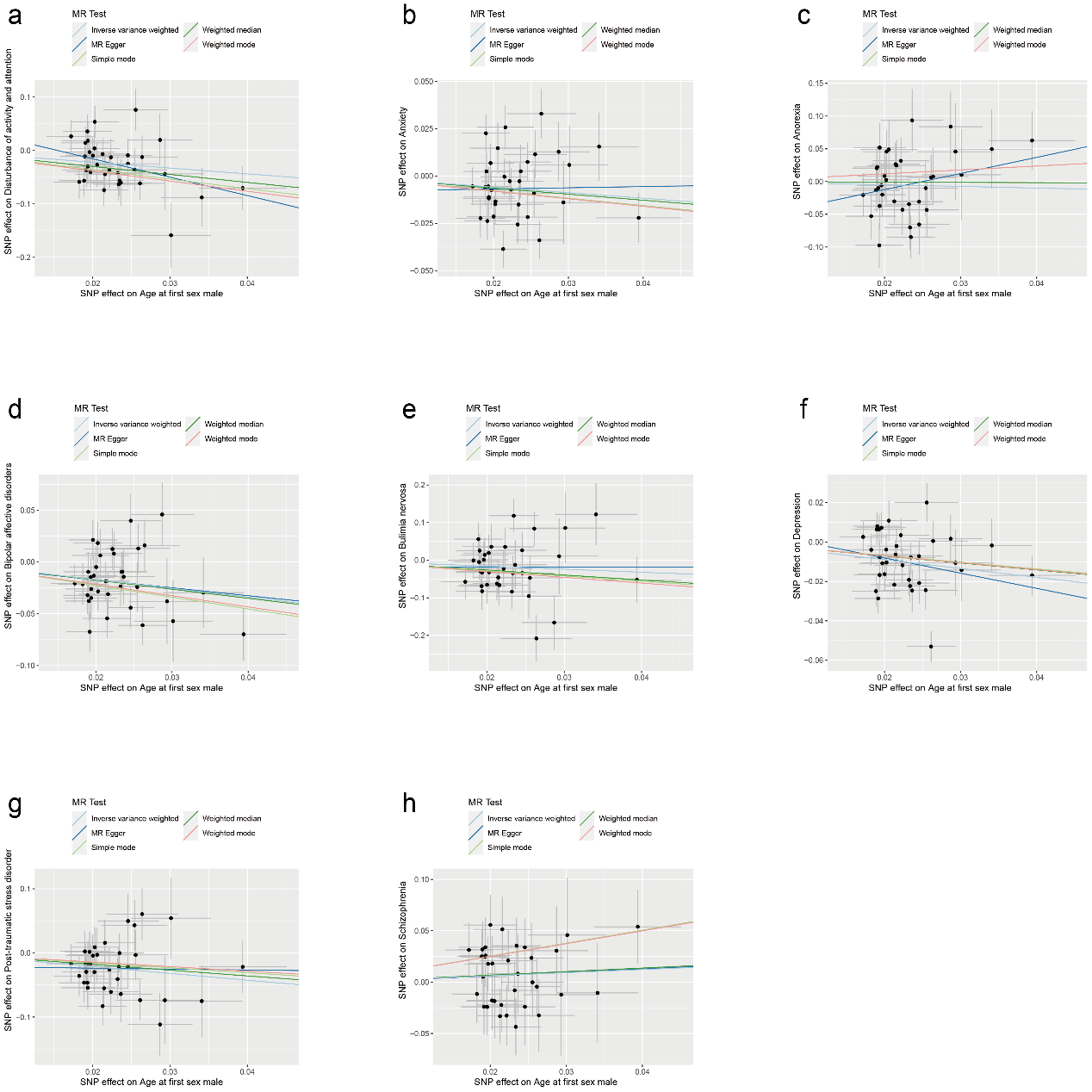 |
| Figure S14. Funnel plot of male AFS on mental disorders  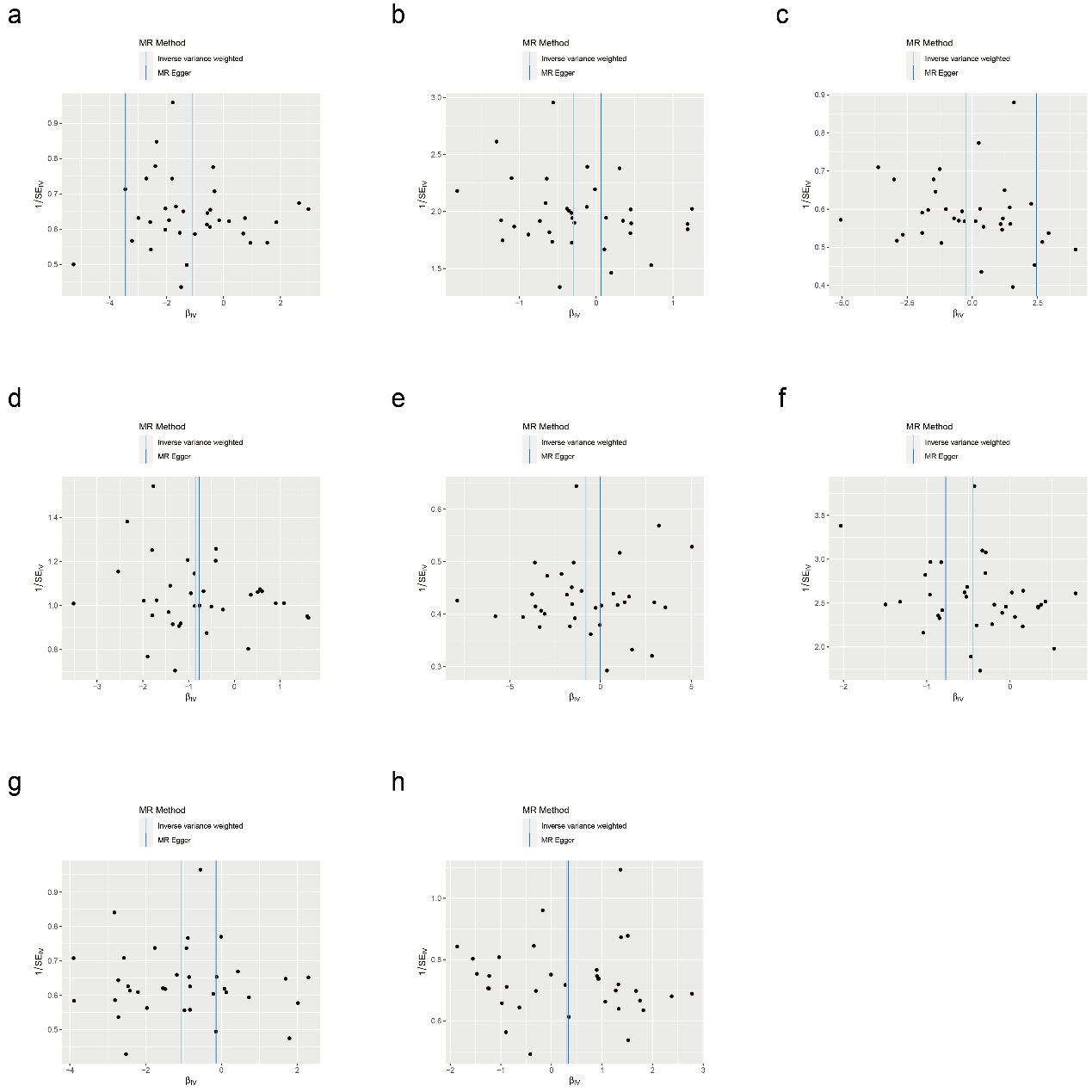 |
| Figure S15. Leave-one-out plot of male AFS on mental disorders  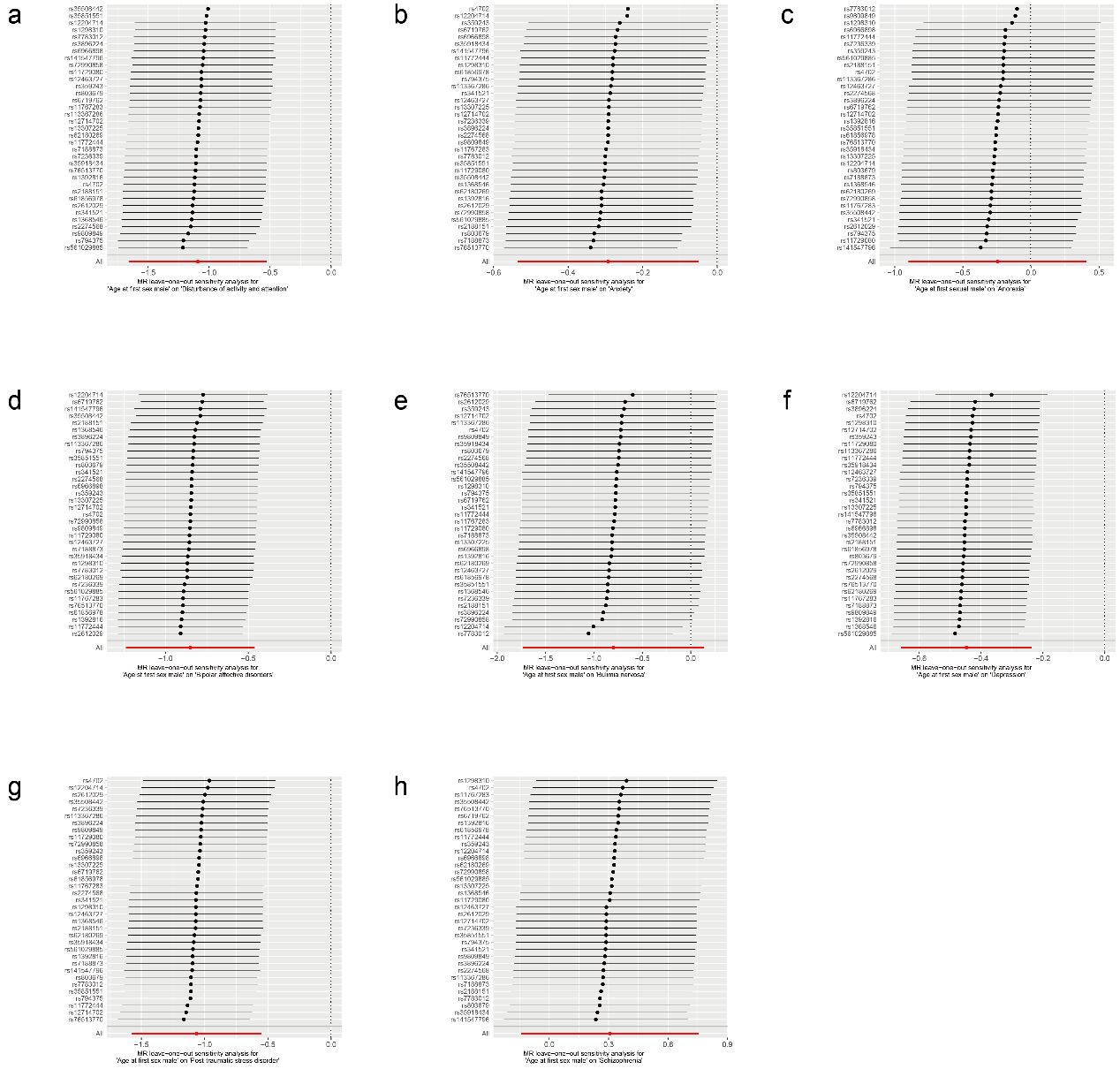 |
| Figure S16. Forest plot of male AFS on mental disorders  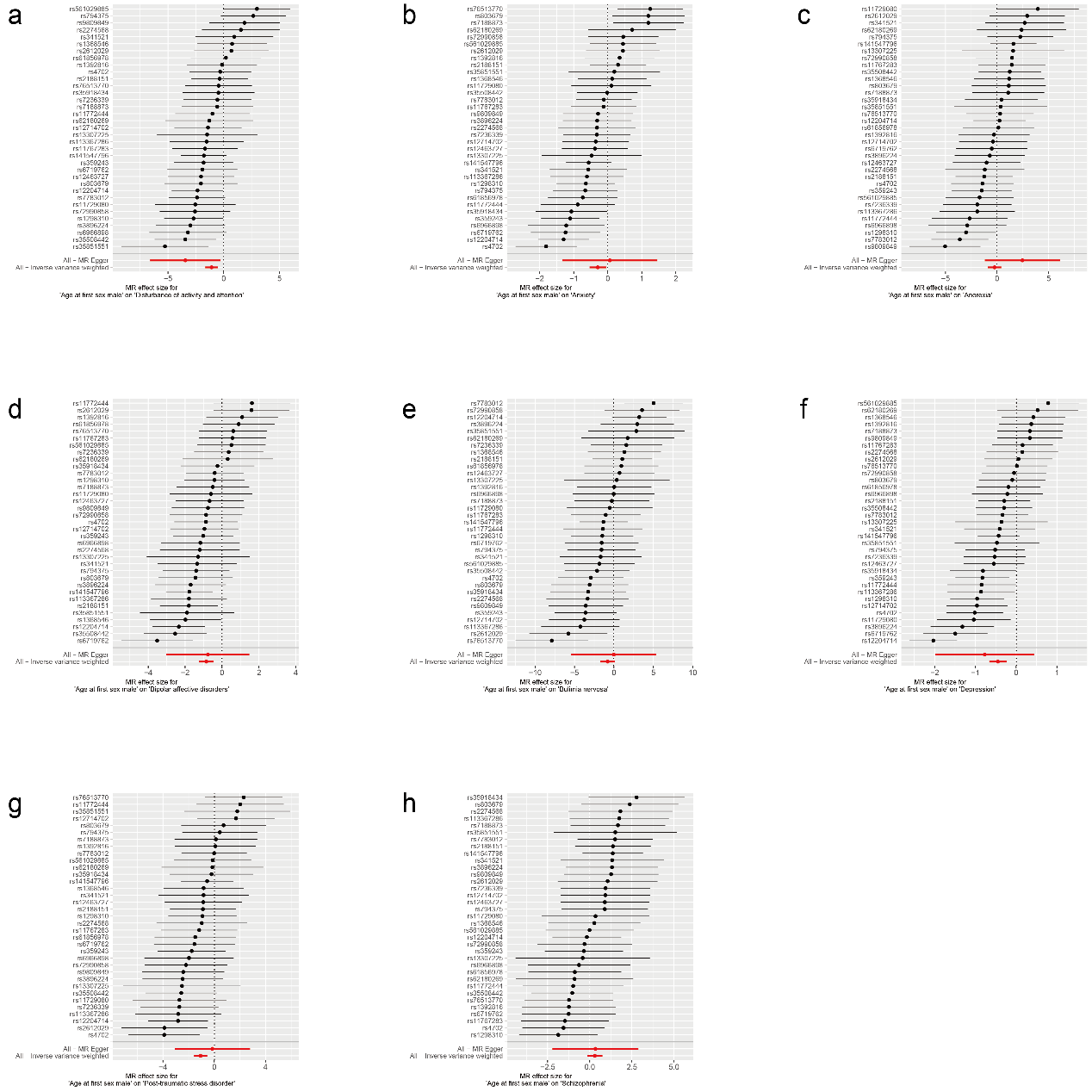 |
| Figure S17. Scatter plot of female NSP on mental disorders  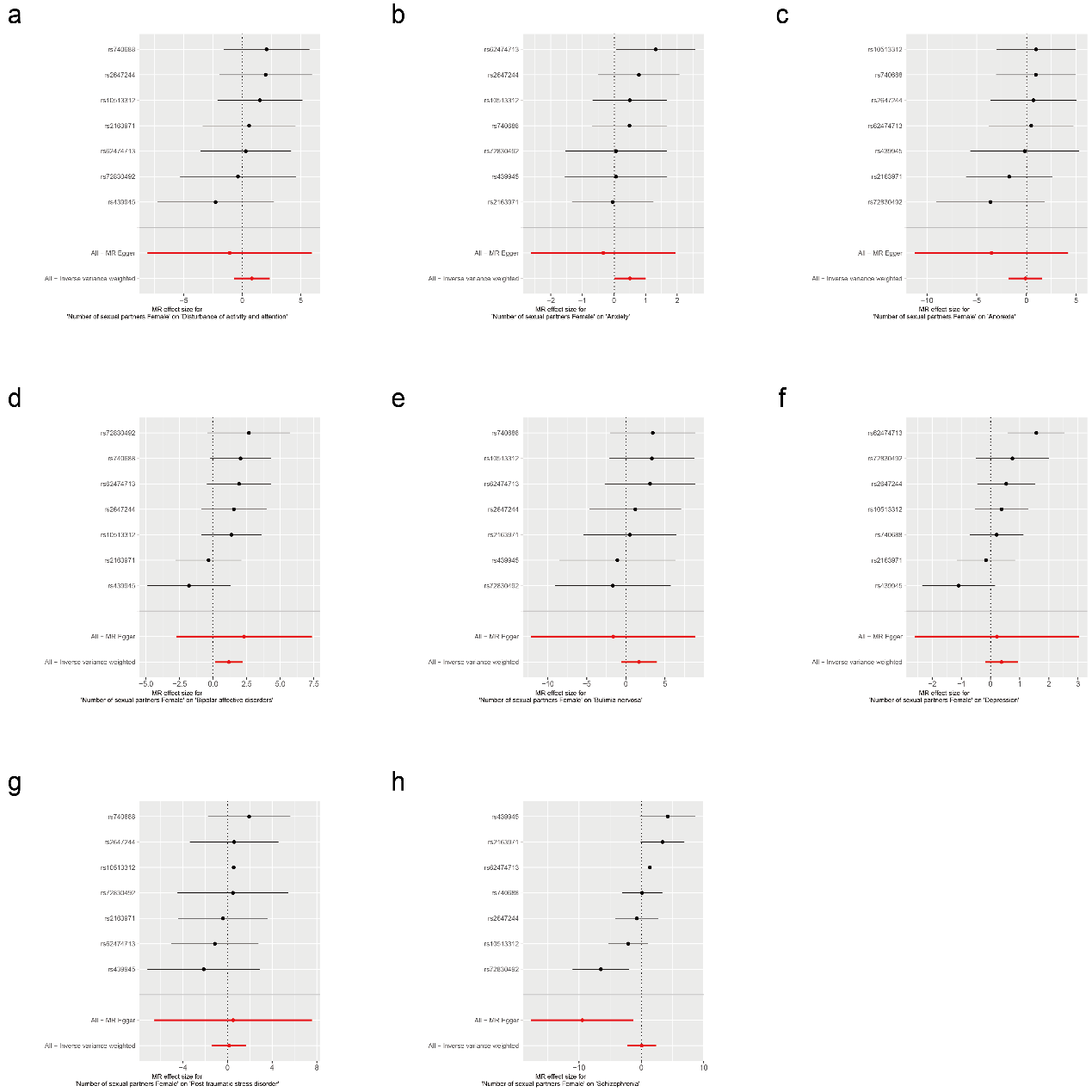 |
| Figure S18. Funnel plot of female NSP on mental disorders  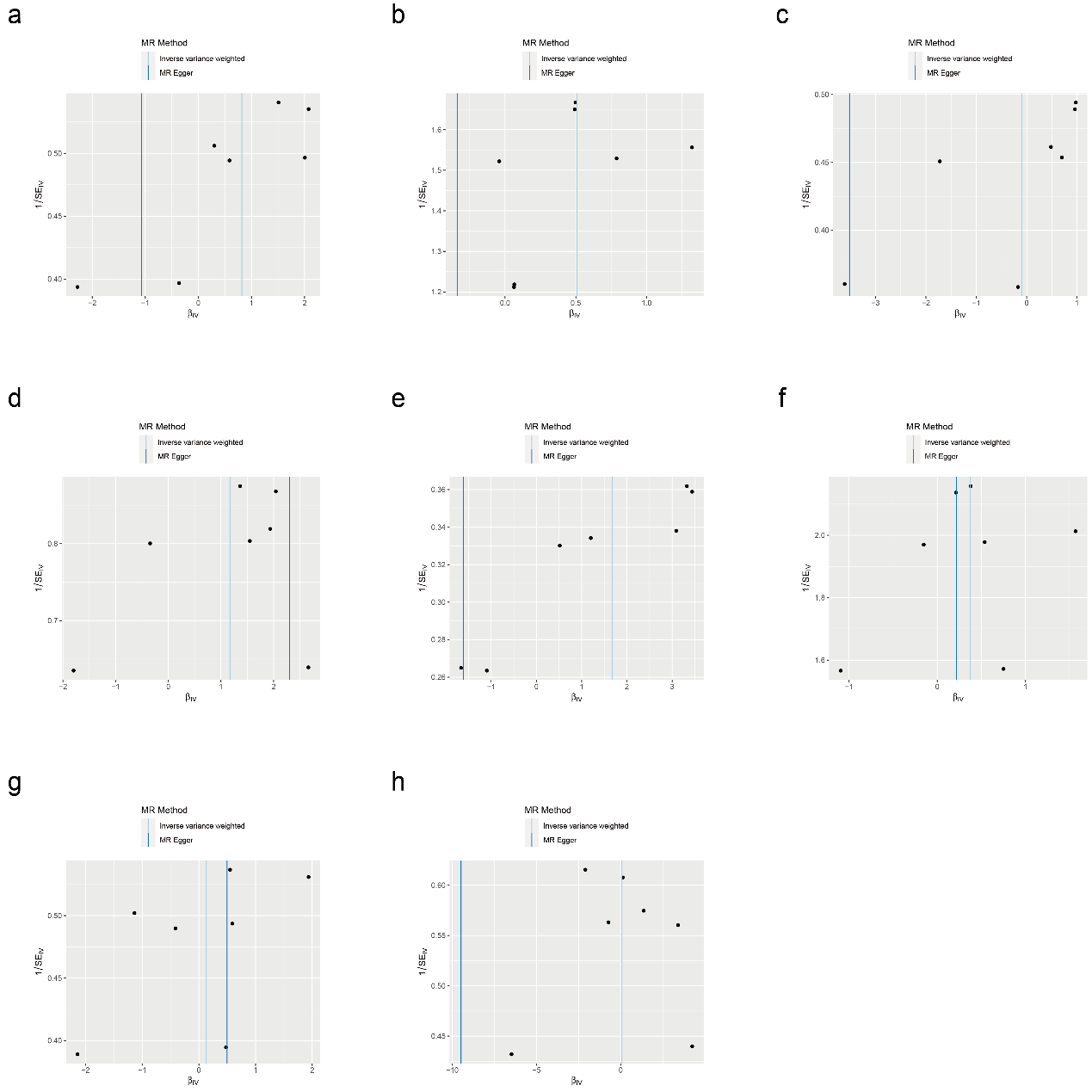 |
| Figure S19. Leave-one-out plot of female NSP on mental disorders  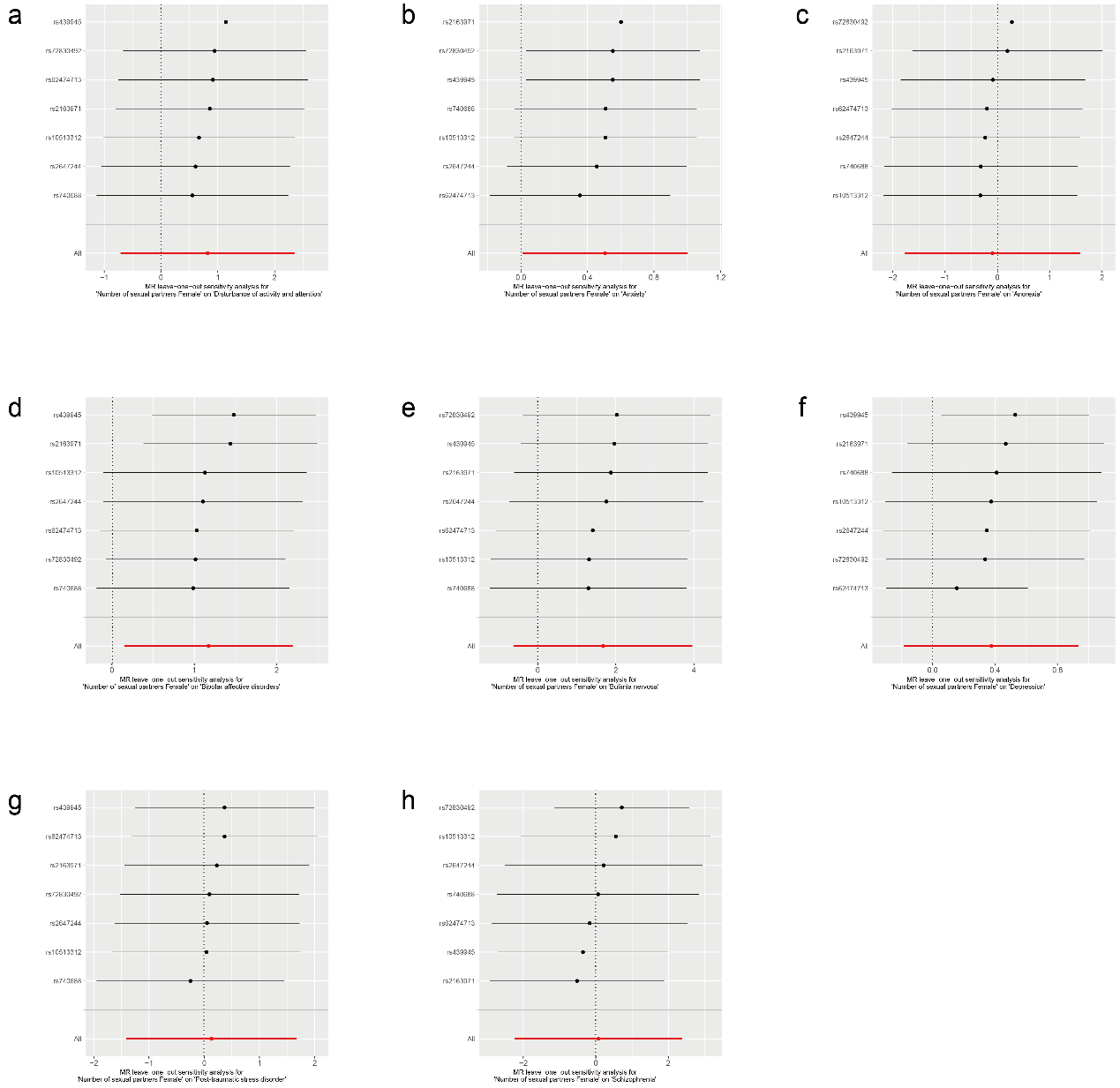 |
| Figure S20. Forest plot of female NSP on mental disorders  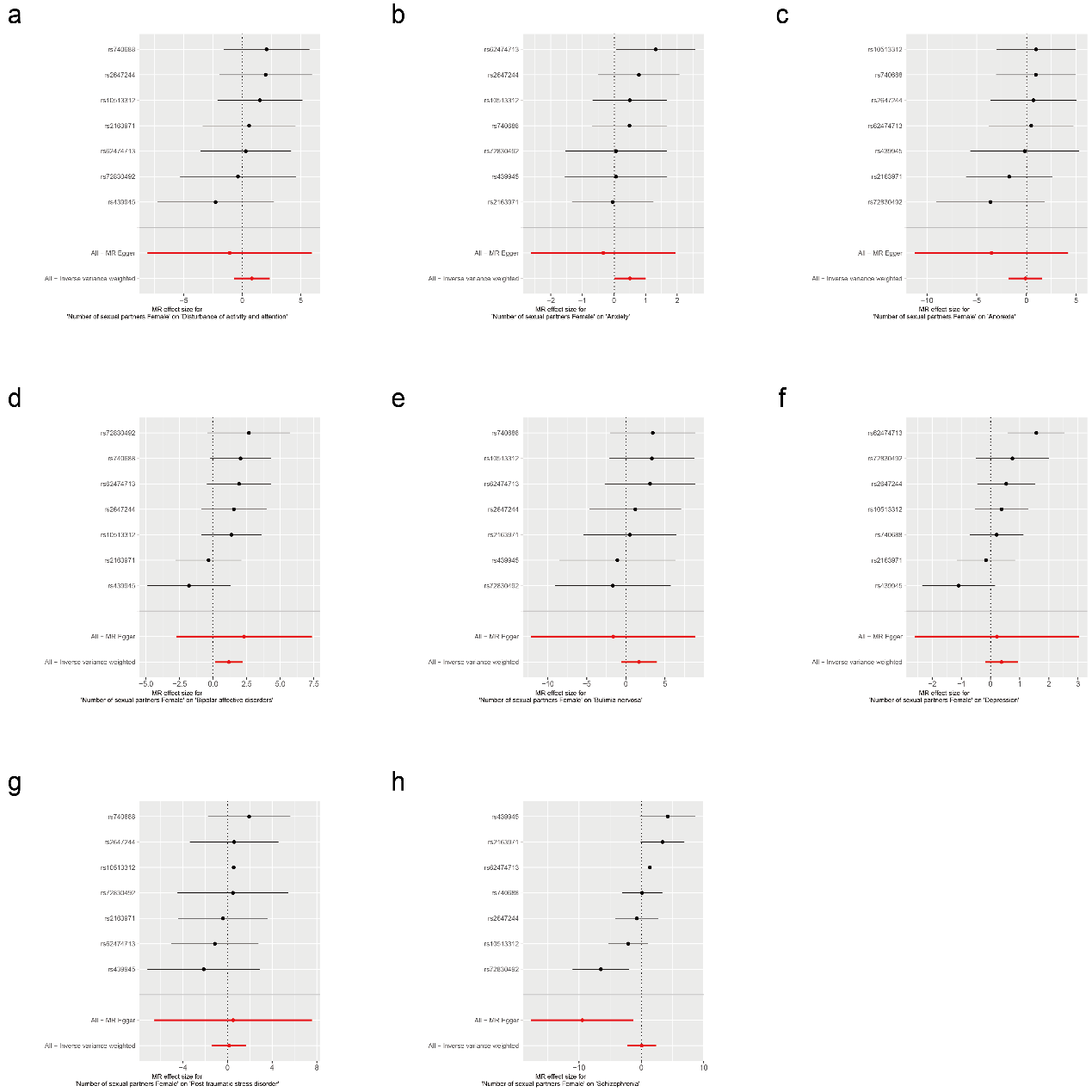 |
| Figure S21. Scatter plot of male NSP on mental disorders  **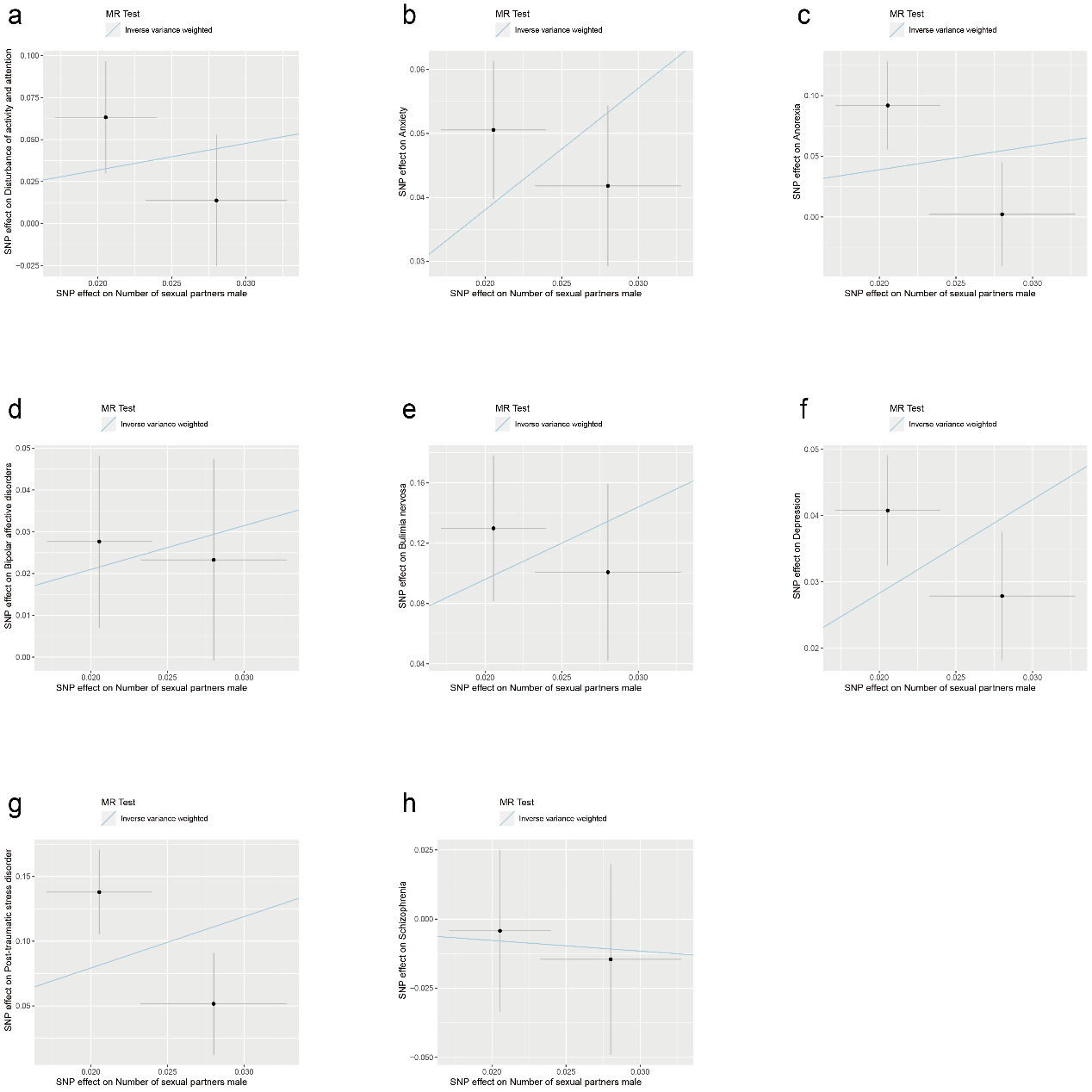** |
| Figure S22. Funnel plot of male NSP on mental disorders  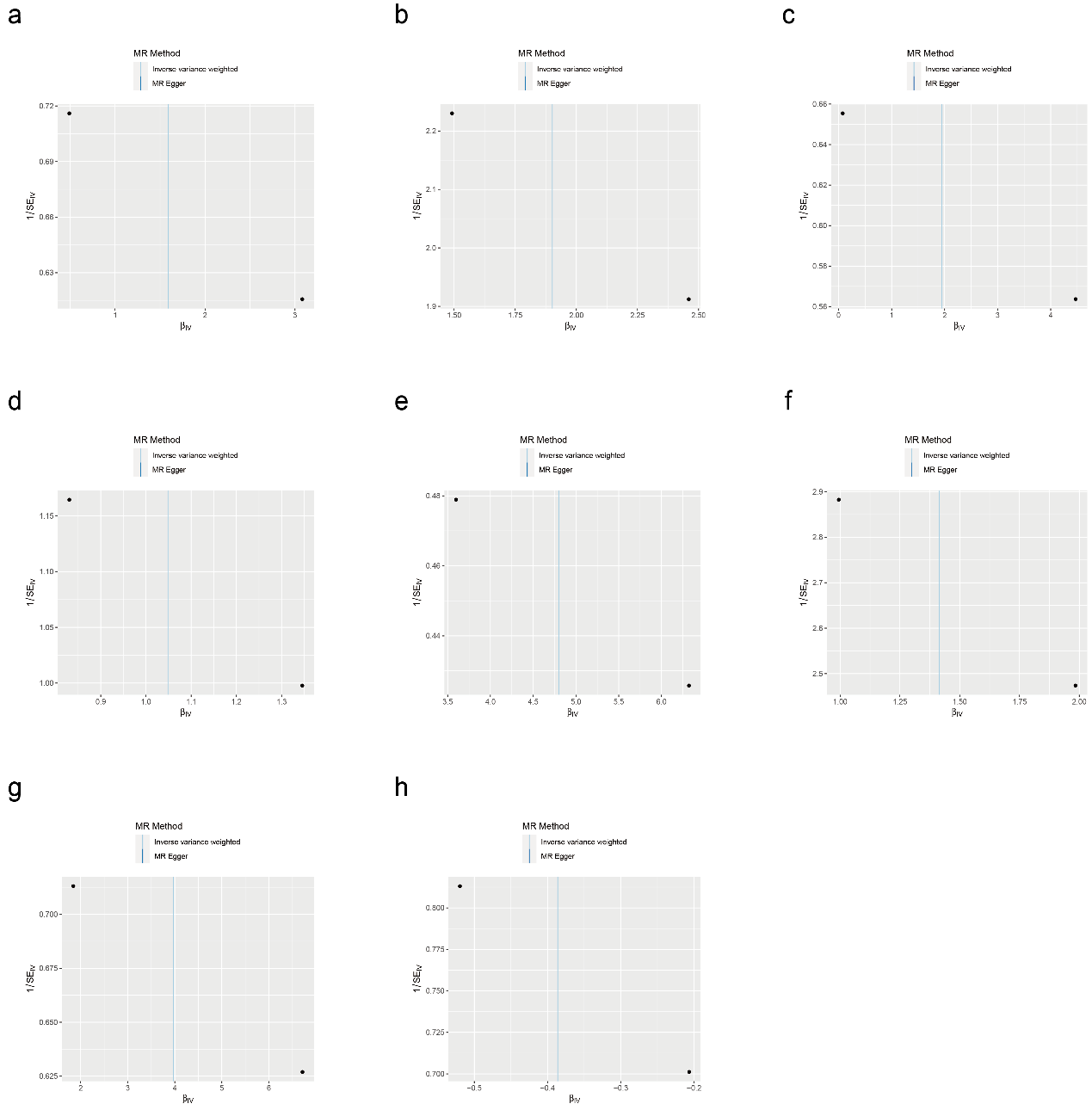  Figure S23. Forest plot of male NSP on mental disorders |


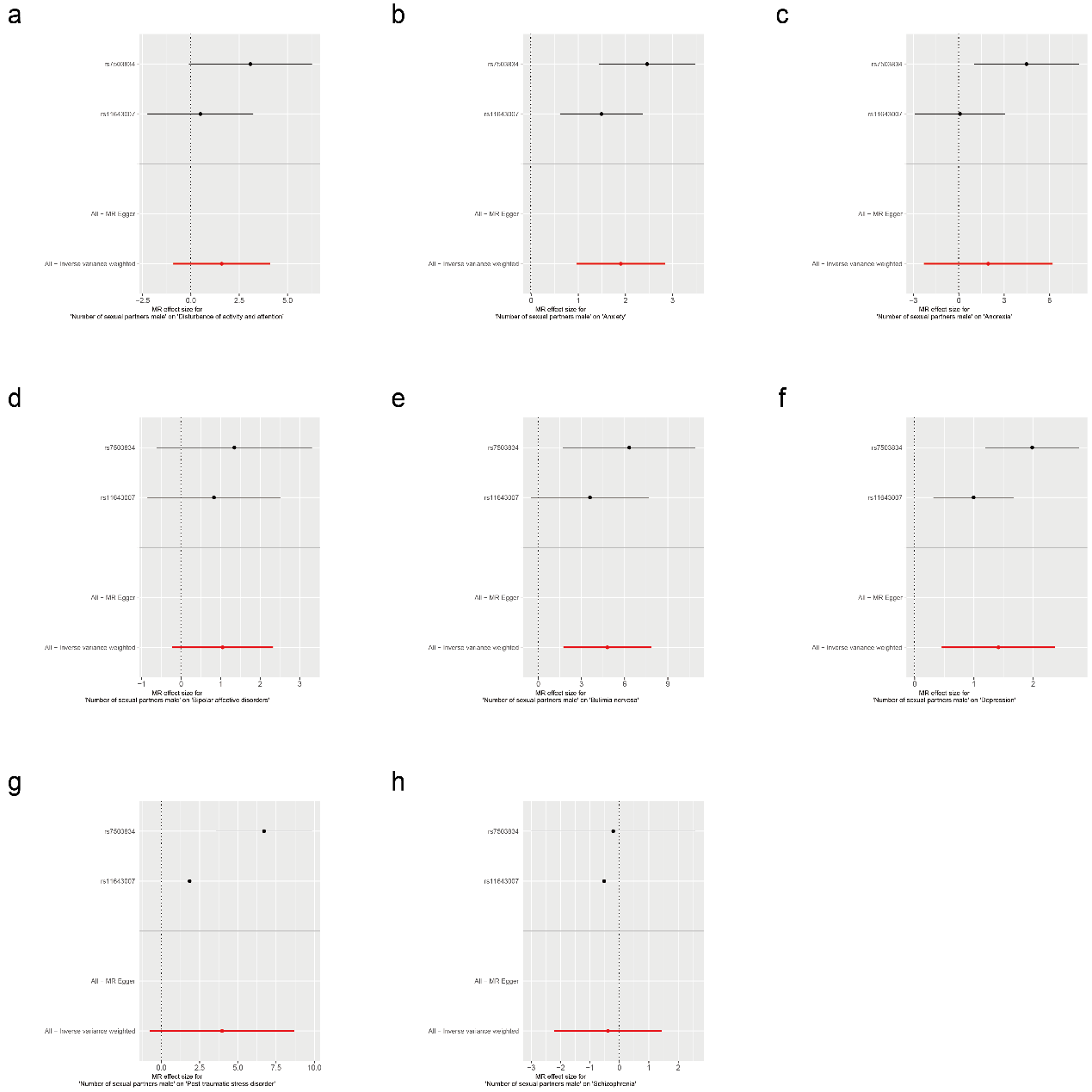


(a-h): (a) disturbance of activity and attention; (b) anxiety disorders; (c) anorexia; (d) bipolar affective disorders; (e) bulimia nervosa; (f)depression; (g) Post-traumatic stress disorder; (h) Schizophrenia. AFS: age at first sex; NSP: number of sexual partners.

Figure S24. The results of multivariable logistic regression analyses in NHANES
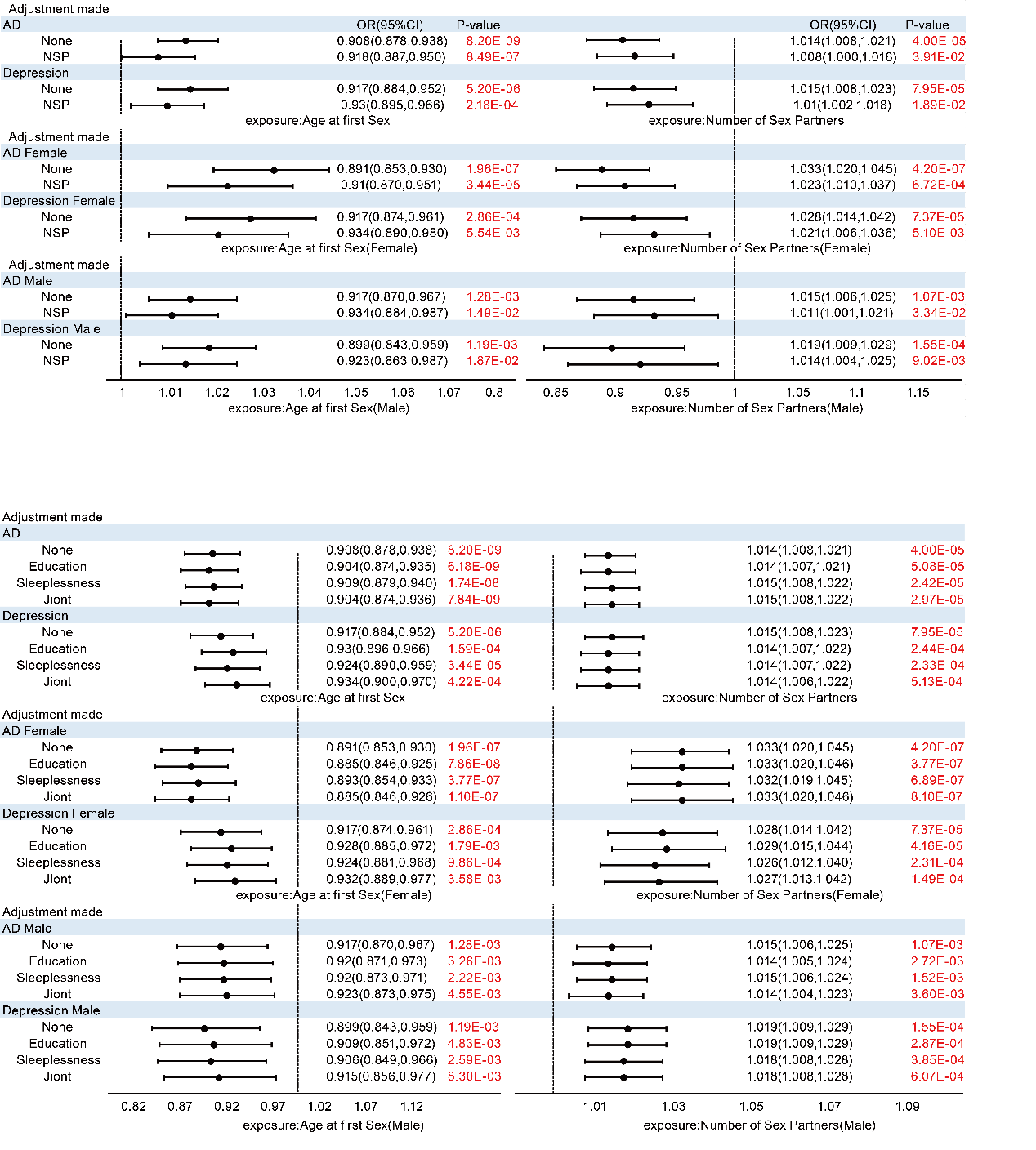

Supplement: Supplementary file 2 — Figure S1: Scatter plot of AFS on mental disorders. Figure S2: Funnel plot of AFS on mental disorders. Figure S3: Leave‐one‐out plot of AFS on mental disorders. Figure S4: Forest plot of AFS on mental disorders. Figure S5: Scatter plot of NSP on mental disorders. Figure S6: Funnel plot of NSP on mental disorders. Figure S7: Leave‐one‐out plot of NSP on mental disorders. Figure S8: Forest plot of NSP on mental disorders. Figure S9: Scatter plot of female AFS on mental disorders. Figure S10: Funnel plot of female AFS on mental disorders. Figure S11: Leave‐one‐out plot of female AFS on mental disorders. Figure S12: Forest plot of female AFS on mental disorders. Figure S13: Scatter plot of male AFS on mental disorders. Figure S14: Funnel plot of male AFS on mental disorders. Figure S15: Leave‐one‐out plot of male AFS on mental disorders. Figure S16: Forest plot of male AFS on mental disorders. Figure S17: Scatter plot of female NSP on mental disorders. Figure S18: Funnel plot of female NSP on mental disorders. Figure S19: Leave‐one‐out plot of female NSP on mental disorders. Figure S20: Forest plot of female NSP on mental disorders. Figure S21: Scatter plot of male NSP on mental disorders. Figure S22: Funnel plot of male NSP on mental disorders. Figure S23: Forest plot of male NSP on mental disorders. Figure S24: The results of multivariable logistic regression analyses in NHANES. [file CNS-32-e70861-s001.docx]
